# Evidence for Genotype-Specific Optimal Blood Lead Levels for Cancer Risk: *MKI67* rs11016073 and *APOB* rs1367117 in a Female Prospective Cohort

**DOI:** 10.3390/ijms27052317

**Published:** 2026-03-01

**Authors:** Krzysztof Lubiński, Wojciech Marciniak, Róża Derkacz, Adam Kiljańczyk, Milena Kiljańczyk, Marcin R. Lener, Sandra Pietrzak, Cezary Cybulski, Tadeusz Dębniak, Tomasz Huzarski, Wojciech Kluźniak, Tadeusz Sulikowski, Jan Lubiński, Rodney J. Scott, Jacek Gronwald

**Affiliations:** 1 International Hereditary Cancer Center, Department of Genetics and Pathology, Pomeranian Medical University in Szczecin, ul. Unii Lubelskiej 1, 71-252 Szczecin, Poland; krzychul123456789@gmail.com (K.L.); adam.kiljanczyk@pum.edu.pl (A.K.); milena.matuszczak@pum.edu.pl (M.K.); marcinlener@poczta.onet.pl (M.R.L.); sandra.pietrzak@pum.edu.pl (S.P.); cezarycy@pum.edu.pl (C.C.); debniak@pum.edu.pl (T.D.); huzarski@pum.edu.pl (T.H.); wojciech.kluzniak@pum.edu.pl (W.K.); lubinski@pum.edu.pl (J.L.); 2 Read-Gene, Grzepnica, ul. Alabastrowa 8, 72-003 Grzepnica, Poland; wojciech.marciniak@read-gene.com (W.M.); roza.derkacz@gmail.com (R.D.); 3 Department of Diagnostic Imaging and Interventional Radiology, Pomeranian Medical University Hospital No. 1, 70-252 Szczecin, Poland; 4 Department of Clinical Genetics and Pathology, University of Zielona Góra, ul. Zyty 28, 65-046 Zielona Góra, Poland; 5 Department of General, Minimally Invasive, and Gastroenterological Surgery, Pomeranian Medical University in Szczecin, 71-252 Szczecin, Poland; tadeusz.sulikowski@pum.edu.pl; 6 Priority Research Centre for Cancer Research, Innovation and Translation, Hunter Medical Research Institute, Newcastle, NSW 2308, Australia; rodney.scott@newcastle.edu.au; 7 School of Biomedical Sciences and Pharmacy, Faculty of Health and Medicine, University of Newcastle, Newcastle, NSW 2308, Australia; 8 Division of Molecular Medicine, Pathology North, John Hunter Hospital, Newcastle, NSW 2305, Australia

**Keywords:** Pb, cancer risk, prospective study, non-occupational exposure, carcinogen, *APOB* rs11016073, *MKI67* rs1367117

## Abstract

This study’s aim was to clarify the regulatory roles of the *MKI67* rs11016073 and *APOB* rs1367117 polymorphisms in the relationship between blood Pb levels and cancer risk. Blood Pb concentrations were measured using inductively coupled plasma mass spectrometry, and genotyping was performed by real-time PCR with TaqMan probes. Cancer incidence was assessed during a mean follow-up of six years and two months. During follow-up, 210 incident cancers were diagnosed among 2782 women. Pb exposure was categorized into quartiles (Q1: <9.44 µg/L; Q2: 9.44–12.58 µg/L; Q3: 12.59–17.16 µg/L; Q4: >17.16 µg µg/L). The association between Pb levels and cancer risk was strongly genotype dependent. Women carrying *APOB* non-GG and *MKI67* non-AA genotypes exhibited the lowest breast cancer risk at the highest Pb levels (Q4), whereas carriers of *APOB* GG and *MKI67* AA showed the lowest risk at the lowest Pb levels (Q1). Age-stratified analyses further demonstrated genotype-specific differences in optimal Pb exposure ranges, particularly for breast cancer. Cancer risk associated with Pb exposure is not uniform but depends on genetic background. These findings identify genotype-specific optimal blood Pb levels and suggest that incorporation of *MKI67* and *APOB* genotyping may improve risk stratification and interpretation of non-linear Pb–cancer associations.

## 1. Introduction

Lead (Pb) is a ubiquitous environmental contaminant, with exposure levels varying substantially depending on geographic location and industrial activity. According to the International Agency for Research on Cancer (IARC), inorganic Pb is classified as probably carcinogenic to humans (Group 2A), whereas organic Pb is currently classified as Group 3 (unclassifiable) [1].

Major sources of environmental Pb contamination include mining, smelting, manufacturing, and recycling activities [2,3,4,5]. In addition, dietary intake via vegetables, cereals, fruits, and other food products represents an important non-occupational exposure route, primarily due to contamination from industrial dust and exhaust emissions [3,6]. Although occupational exposure remains the predominant source of Pb in adults, non-occupational exposure is widespread and contributes to measurable blood Pb levels in the general population.

Pb exerts its biological toxicity through multiple mechanisms, including interference with calcium-dependent processes and substitution for essential metals, resulting in structural and functional alterations of enzymes and regulatory proteins [7,8,9,10]. Proposed mechanisms of Pb carcinogenicity include direct DNA damage, inhibition of DNA repair and synthesis, generation of reactive oxygen species, and disruption of zinc-dependent transcriptional regulation through zinc finger proteins [11,12,13].

To date, fourteen epidemiological studies have examined the association between blood Pb levels and cancer risk, including both population-based and occupational cohorts. Collectively, these studies suggest that Pb exposure is associated with an increased risk of several malignancies, notably breast cancer, particularly among women below 50 years of age [14,15,16,17,18,19,20,21,22,23,24,25,26,27] [Table S1]. However, the reported exposure–response relationships are heterogeneous, and several studies have described non-linear or non-monotonic patterns that are difficult to reconcile with a uniform carcinogenic effect of Pb.

One potential explanation for these inconsistencies is inter-individual variability in genetic susceptibility to Pb toxicity. Genes involved in cell proliferation and lipid metabolism may modulate Pb-induced carcinogenic processes by influencing DNA damage responses, oxidative stress, and inflammatory pathways.

The *MKI67* gene encodes the proliferation marker Ki-67, which is widely used as an indicator of cellular proliferation and tumor aggressiveness. Pb has been shown to affect *MKI67* transcription and protein expression, suggesting that genetic variation in *MKI67* may modify susceptibility to Pb-related carcinogenesis. The *APOB* gene plays a central role in lipid transport and oxidative processes, and *APOB* polymorphisms have been implicated in cancer risk through mechanisms involving inflammation, oxidative stress, and lipid dysregulation.

Therefore, the present study was designed to clarify the regulatory roles of the *MKI67* rs11016073 and *APOB* rs1367117 polymorphisms in the relationship between blood Pb levels and cancer risk. By integrating genetic stratification with exposure assessment, this study aims to determine whether genotype-specific susceptibility contributes to the heterogeneous and non-linear associations observed in previous Pb epidemiological studies.

## 2. Results

**Figure 1:**
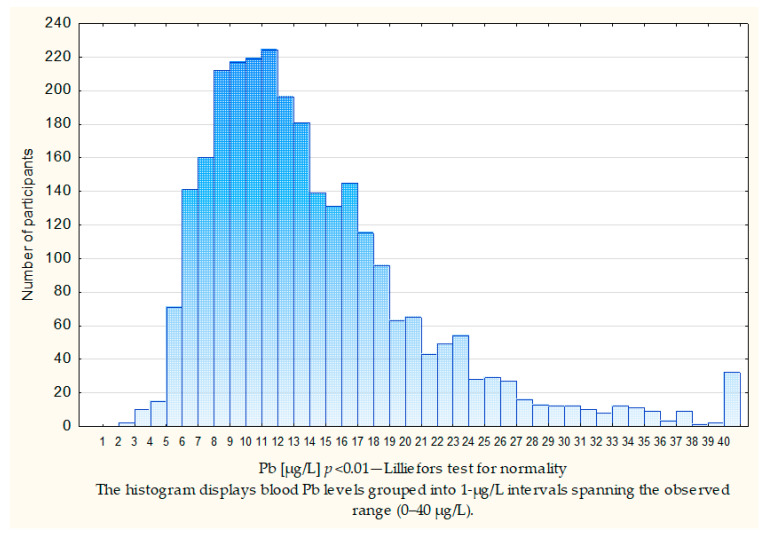
The distribution of the blood Pb levels in all patients.

The final cohort included 2782 cancer-free women. Each woman provided a single sample from which their blood Pb levels were measured. The mean subject age at the time of blood extraction was 52 years and 5 months (range 40–83 years). Table 1 presents the patients characteristics of the women enrolled in the study. The average blood Pb level was 14.38 µg/L. The mean Pb levels in the blood for different groups are shown in Table 1. Blood Pb concentrations were elevated in women over the age of 50 in comparison to those below this age (*p* < 0.01). Furthermore, these levels were augmented in current and former smokers (*p* < 0.01). Figure 1 illustrates the distribution of blood Pb levels on the entire study cohort.

**Figure 2:**
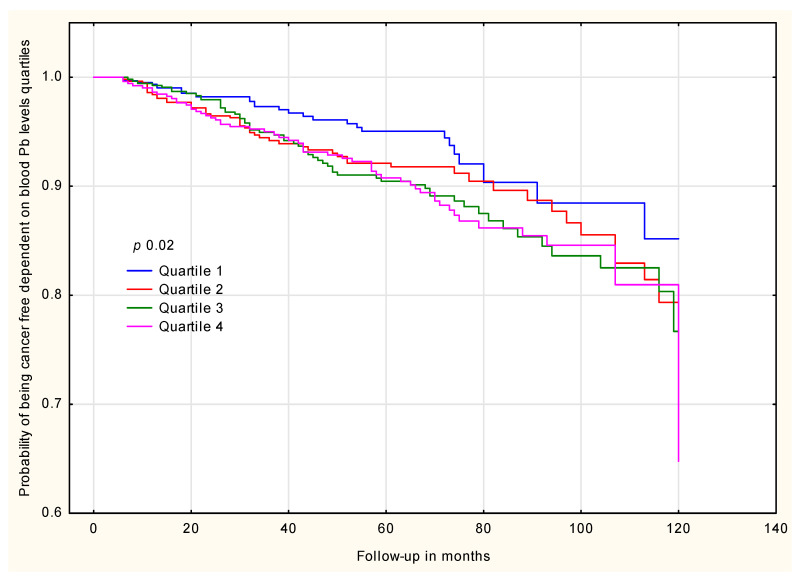
Ten-year cancer risk by blood Pb levels.

Women were observed for a mean period of 6 years and 2 months (range 0.5 to 13.5 years). During the follow-up, 210 cancer incidents were documented among which 106 were breast cancers and 104 other cancers [Table 2]. The display of the ten-year cumulative cancer risk by blood Pb quartiles is shown in Figure 2.

Four groups (quartiles) of similar size of unaffected females were used to divide the 2782 participating women according to their total blood Pb level. The cut-off levels to define the quartiles were (Q1) <9.44 µg/L, (Q2) 9.44–12.58 µg/L, (Q3) 12.59–17.16 µg/L, and (Q4) >17.16 µg/L. Analysis was conducted on the entire group and on subgroups: below 50 years of age, above 50 years of age, with any cancers (including breast cancer).

### 2.1. The Whole Group—Any Cancer

The results on the whole group without genotyping for any cancer were not statistically significant as determined by multivariate analysis [Table S2]. The results, including genotype analysis, showed moderate improvement, with some statistically significant evidence [Tables S3–S6].

### 2.2. The Whole Group—Breast Cancer

Results from the breast cancer risk group irrespective of age without genotypes showed no statistical significance [Table S7]. Inclusion of the genotype analysis revealed a statistically significant correlation between women with the *MKI67* non-AA genotype [Table 3]. This result showed that the lowest breast cancer risk was in Q4 compared to Q2 (HR = 4.80; 95% CI: 1.75–13.13; *p* = 0.0022).

The additional analyses of breast cancer risk across Pb quartiles for the *MKI67* AA genotype and both *APOB* genotype groups (non-GG and GG) are presented in Tables S8–S10. Although several associations reached statistical significance in these subgroups, they did not meet the predefined criteria for inclusion in the main analysis (HR > 4 and *p* < 0.01). Therefore, these results are reported in the Supplementary Materials for completeness.

### 2.3. The Subgroup of Women Older than 50 Years of Age and Any Cancer

The results of the subgroup of all cancers diagnosed in women above 50 years of age without genotyping showed no statistically significant association [Table S11]. Adding genotype information to the analysis also did not reveal any significant association [Tables S12–S15].

### 2.4. The Subgroup of Women Older than 50 Years of Age Diagnosed with Breast Cancer

Breast cancer risk in this group of women in the absence of genotype analysis was not associated with any specific risk of disease [Table S16]. Incorporation of genotypes analysis revealed statistical associations with Pb depending on the genotyping results; women with *MKI67* non-AA had the lowest cancer risk in Q4 (Q4 vs. Q2) (HR = 5.78; 95% CI: 1.52–21.90; *p* = 0.009) [Table 4]. In contrast, women with the *MKI67* AA genotype had the highest cancer risk in Q2 (Q2 vs. Q4; HR = 4.26; 95% CI: 1.48–12.29; *p* = 0.007) [Table 5]. Additional results are presented in supplementary tables [Tables S17 and S18].

### 2.5. The Subgroup of Women Less than 50 Years of Age and Any Cancer

We found a statistically significant association between Pb levels and cancer risk (see Table S19), where the lowest blood Pb levels appeared to be associated with the lowest cancer risk. The strongest correlation in this group was observed in women with *APOB* non-GG genotype in quartile 1 which had over four-fold lower risk of any cancer compared to women in quartile 3 (HR = 4.57; 95% CI: 1.57–13.27; *p* = 0.005) [Table 6]. The remaining results for any cancer risk with genotypes analyses are presented in Supplementary Tables [Tables S20–S22].

### 2.6. The Subgroup of Women Under the Age of 50 Years and Their Breast Cancer Risk

Additional subgroup analyses for both *MKI67* genotypes (non-AA and AA) and for both *APOB* genotypes (non-GG and GG) are summarized in supplementary [Tables S23–S27]. Some associations were statistically significant; however, they did not satisfy the predefined thresholds for inclusion in the main results section (HR > 4 and *p* < 0.01).

### 2.7. Combined Genotypes

The following combinations of genotypes: *APOB* non-GG or/and *MKI67* non-AA; *APOB* GG or/and *MKI67* AA; *APOB* non-GG or/and *MKI67* AA; *APOB* GG or/and *MKI67*non-AA were assessed for their relationship to disease. The analysis revealed overall cancer risk for the entire group; cancer risk for women over 50 years of age; women 50 years of age or younger; breast cancer risk irrespective on age; breast cancer risk for women over 50 years of age; and breast cancer risk for women at or below 50 years of age.

The results for any cancer risk irrespective of age are shown in Supplementary [Tables S28–S35].

For the women with *APOB* non-GG and *MKI67* non-AA genotypes the reference blood Pb level with the lowest cancer risk was quartile 4 comparing Q4 vs. Q2 (HR = 15.32; 95% CI: 1.96–119.60; *p* = 0.009) [Table 7]. In contrast, women with the *APOB* GG and MKI AA genotypes had the lowest cancer risk in Q1 comparing to Q4 had an almost six-fold lower breast cancer risk irrespective on age [Table 8]. No other results for breast cancer risk irrespective on age were significant and are presented in Supplementary [Tables S36–S41].

The results for any cancer risk in women above 50 years of age did not show any statistical significance regardless of genotype [Tables S42–S49].

Of note we did observe an association with breast cancer risk in women above 50 years of age carrying the *APOB*GG and *MKI67*AA genotype. Women with lower Pb levels (quartiles 1–3) had more than a four-fold lower cancer risk than women in Q4 (HR = 4.43; 95% CI: 1.68–11.68; *p* = 0.002) [Table 9]. There were no other significant results for breast cancer risk in these women (data presented in Supplementary Materials [Tables S50–S56]).

For cancer risk in women below 50 years of age, there were no significant results [see Supplementary Tables S57–S64]. Similar results were observed for women with breast cancer risk below 50 years of age [Tables S65–S72].

## 3. Discussion

The central finding of this study is that the carcinogenic potential of Pb is strongly modified by genetic background.

At first glance, some of the present findings may appear to challenge the prevailing view of lead as a uniformly carcinogenic exposure. However, rather than contradicting existing evidence, results provide a potential explanation for the non-linear exposure–response relationships reported in previous studies. Importantly, the inclusion of genetic stratification should not be viewed as a controversial departure from existing evidence. Several epidemiological studies have already reported non-monotonic exposure–response relationships for Pb, in which intermediate exposure quartiles were associated with a higher relative risk than either lower or higher exposure categories. In some reports, the highest exposure quartile exhibited a substantially elevated risk compared with the lowest quartile, yet the relative increase in risk was smaller than that observed in the intermediate quartile.

Such patterns are difficult to reconcile with a uniform biological response to Pb exposure. Our findings suggest that genetic heterogeneity may provide a mechanistic explanation for these observations, whereby genotype-specific susceptibility thresholds shape the apparent non-linearity of the exposure–response relationship at the population level.

Without genotypic stratification, no association between Pb levels and cancer risk was observed in women aged ≥50 years. In contrast, incorporation of genetic data revealed distinct subgroups in which Pb exposure was associated with either increased or neutral cancer risk.

The most pronounced modification effect was observed for the *MKI67* genotype. Women carrying the *MKI67* non-AA genotype exhibited the highest tolerable blood Pb levels without an increase in cancer risk, whereas carriers of the AA genotype appeared to benefit from lower Pb levels. These findings suggest that Pb exposure thresholds relevant to carcinogenesis are not uniform but depend on proliferative signaling pathways influenced by *MKI67*.

The biological plausibility of this observation is supported by experimental data demonstrating that Pb exposure directly affects *MKI67* transcription and Ki-67 protein expression. Ki-67 is a well-established marker of cellular proliferation and tumor aggressiveness, and its altered regulation may modulate the carcinogenic response to Pb exposure [28,29,30,31,32,33].

In contrast, the modifying effect of the *APOB* genotype appears to be indirect. *APOB* polymorphisms influence lipid metabolism, oxidative stress, and inflammatory pathways, all of which may contribute to Pb-related carcinogenic mechanisms. In the present cohort, women with the *APOB* non-GG genotype appeared to benefit from lower Pb levels, particularly in the context of Pb toxicity mitigation [34,35,36,37,38,39,40,41].

Age-stratified analyses further support the genotype-dependent nature of Pb carcinogenicity. Consistent with our previous studies, an elevated hazard ratio was primarily observed in women below 50 years of age. However, genotypic stratification substantially refined risk estimates, allowing identification of subgroups at both increased and decreased risk across age categories.

The major strength of this study lies in the magnitude of the observed hazard ratios and the robustness of the statistical evidence. Nevertheless, certain findings—such as similar risk estimates observed in extreme Pb quartiles for specific genotype combinations—should be interpreted with caution and it cannot be excluded that they are attributable to limited case numbers within these strata. Validation in larger cohorts is therefore essential.

Several limitations must be acknowledged. Blood Pb concentration was measured at a single time point, occupational exposure history was self-reported, and socioeconomic status was not formally assessed. Although samples were processed in a blinded manner and stored under identical conditions, residual confounding cannot be excluded.

In conclusion, these findings suggest that Pb-related cancer risk cannot be adequately assessed without consideration of genetic susceptibility. Incorporation of targeted genotyping may enable stratification of individuals into distinct risk categories and facilitate more personalized approaches to Pb exposure management and cancer prevention. Further studies in independent populations are warranted to validate these observations.

## 4. Materials and Methods

### 4.1. Study Group

The study’s subjects were 2782 female patients from the Cancer Genetics Outpatient Clinics aged 40 years or more. We restricted the study population to women aged 40 years or older because, below this age, the incidence of cancer—particularly breast cancer and other tumors—remains very low in the general population. The age threshold was therefore chosen to ensure a sufficient number of events during follow-up and to allow meaningful risk estimation. All women in this study underwent genetic testing for at least three BRCA1 founder pathogenic variants (PVs) that are the most frequent in Poland (c.5266dupC, c.181T>G, and c.4035delA) at the Pomeranian Medical University in Szczecin between September 2010 and March 2024. Over 90% of all inherited predispositions to breast cancer in Poland that are associated with *BRCA1* are accounted for by these three founder PVs [42]. None of the participants had been diagnosed with cancer at the time of enrolment. All women who took part gave written informed consent to participate in the study and agreed to provide a blood sample for the study. In the ensuing period, patients were contacted at annual visits for the purpose of routine monitoring. The investigation was carried out in compliance with the Helsinki Declaration with the permission of the Ethics Committee of Pomeranian Medical University in Szczecin. At the initial outpatient visit, a blood sample was collected for genetic testing. For future studies, an additional 10-mL sample of whole blood was additionally gathered and kept at −80 °C. A detailed questionnaire was provided to all women, which comprised a comprehensive review of the patient’s medical history. Any woman who reported a family history of cancer (at any age), smoking status, hormone use, and personal medical history (including oophorectomy), were diagnosed with a founder *BRCA1* PV or had a cancer diagnosis were excluded from the study. Women aged below 40 years of age and those who were exposed to Pb in their workplace were also excluded from the study.

### 4.2. Measurement of Blood Pb

All study participants had 10 mL of peripheral blood collected into a vacutainer tube containing sodium ethylenediaminetetraacetic acid (EDTA) and then stored at −80 °C until analysis. The subjects of the study were required to fast for a minimum of six hours prior to the collection of their samples. On average, 41 months (range 6–105 months) elapsed between the date of blood collection and the date of Pb measurement. On the day preceding analysis, the samples were thawed at room temperature. Before Pb determination, all samples were gently mixed using vortexing. The single-quadrupole ICP-MS was used to determine the 208 Pb ELAN DRC-e (PerkinElmer, Concord, Toronto, ON, Canada). Oxygen was used as the reaction gas. Before analysis, the ICP-MS was calibrated according to the manufacturer’s instructions. Critical parameters were optimized for maximum sensitivity. The spectrometer was calibrated using an external calibration technique. Daily, the Multi-Element Calibration Standard 3 was used to prepare calibration standards using 10 µg/mL (PerkinElmer Pure Plus, Shelton, CT, USA), and the final levels were reached by diluting it with a blank reagent 0.5 µg/L, 1 µg/L, 2 µg/L, 5 µg/L, and 10 µg/L for Pb-level determination. All correlations for the calibration curves were always greater than 0.999. Matrix matching was used to perform the calibration. The internal standard was rhodium (^103^Rh). The blood sample was subjected to a 40-fold dilution process using a blank reagent as a diluent (70 µL of blood + 2730 µL of blank reagent). High-purity water was used to make the blank reagent (>18 MΩ), TMAH (AlfaAesar, Kandel, Germany), Triton X-100 (PerkinElmer, Shelton, CT, USA), ethanol (Merck, Darmstadt, Germany), rhodium (PerkinElmer, Shelton, CT, USA), and EDTA (Sigma-Aldrich, Leuven, Belgium).

A tetramethylammonium hydroxide solution was used for dilutions due to the specificity of the measurement. The blood components were readily soluble due to the alkaline pH, and no fractions precipitated. To enhance the dispersion of dissolved blood components, Triton T-100, a non-ionic surfactant, was used. The stability of metal ions dissolved in the solution was achieved by adding edetic acid. Ethanol was added to all solutions due to the high content of carbon-containing compounds in the tested sample to eliminate the effect associated with a significant amount of carbon. The following LOD and LOQ values were obtained: 0.137 µg/L and 0.0328 µg/L, respectively.

#### Quality Control

All measurements were tested to ensure that the accuracy and precision were satisfactory, with the use of certified reference material CRM Clincheck Plasmonorm Blood Trace Elements Level 1 (Recipe, Munich, Germany). The recovery rate was between 80 and 105%. The calculated recurrence rate (Cv%) for was below 15%. Two independent external quality assessment schemes have recognized the testing laboratory as a member the LAMP organized by the CDC and the QMEQAS organized by the Institute National de Santé Publique du Québec. Batch effect was controlled by measuring CRM every 90 samples. Moreover, the same procedure was followed when measuring a quality control sample that had been prepared in the lab (QCS2 mean-11.6; SD 0.61; RSD 5.29).

### 4.3. Genotype Selection

In order to identify the right genes interacting with Pb that could impact on the risk of cancer, we selected a series of genes after a PubMed literature search. The criteria on which genes were included for further analysis were any type of gene interactions both direct and/or indirect with Pb and if a given gene had any association with tumour development. After the first stage screening 156 genes were identified. The next step was to find appropriate polymorphisms of selected genes. The search was based on Polinome Genomic Variants Database (300 adults Poles unaffected, from families without cancers and studied by whole exome sequencing). We searched for polymorphisms reported in the literature and bioinformatically, predicted to have a functional impact on the respective protein. Additionally, the selected genotype frequency was in the range of 25–75%. After the second stage of screening, seven genes were chosen that fulfilled the search criteria: *CAT* rs1001179; *CASP9* rs2234723; *GPX1* rs1050450; *APOB* rs1367117; *HRG* rs10770; *HLA-DRB1* rs9281873; *MKI67* rs11016073.

From the seven candidate genes identified in the second screening stage, two (*MK167* and *APOB*) were selected for the present study. These genes were chosen because, in the preliminary screening conducted on 329 case/control samples, their polymorphisms showed the strongest and most consistent associations with any cancer risk. Although one additional gene initially demonstrated a significant association with any cancer risk in the preliminary screen, this association was not confirmed in the full cohort analysis and therefore the gene was excluded from further investigation. Only *MK167* and *APOB* demonstrated statistically meaningful effects that warranted validation in the complete cohort of 2782 women.

### 4.4. Genotype Assessment

Molecular analyses were performed using real-time polymerase chain reaction (RT-PCR) analysis using a LightCycler 480 device from (Roche Diagnostics, Mannheim, Germany). The studies used Taqman molecular probes in a TaqMan Assay mixture containing probes and PCR reaction primers purchased from (Applied Biosystem, Foster City, CA, USA). The *MKI67* rs11016073 and *APOB* rs1367117 analysis system was custom-made. Each analysis was performed in a volume of 5 µL according to the following proportions: 2.5 µL Probe qPCR MMx 2x (Promega Corporation, Madison, WI, USA); 0.125 µL TaqMan Assay 40x (Applied Biosystems); 1.375 µL Water; 1 µL DNA (25 ng/µL). The real-time PCR reactions were performed on the Light Cycler 480 instrument (Roche). The cycling protocol began with an initial denaturation at 95 °C for 10 min, followed by 50–55 cycles consisting of denaturation at 95 °C for 10 s, annealing at 52 °C for 30 s, and extension at 72 °C for 10 s. After amplification, the program included cooling and a color-compensation phase according to the manufacturer’s recommendations (40 °C for 30 s, followed by a brief denaturation and hybridization step at 80 °C). Fluorescence acquisition was carried out continuously during the hybridization step, with temperature increments of 1 °C per cycle where required.

### 4.5. Statistical Analysis

Data on cancer occurrence were obtained from medical files and pathology reports from the hospitals where participants were treated. In addition, active follow-up was conducted through annual telephone contact and regular clinical surveillance at the Hereditary Cancer Centre, including routine screening examinations. All women were included in the study at the time of blood collection, which occurred at or after the age of 40.

Follow-up began on the date of blood draw and continued until the first cancer diagnosis, death from any cause, or the date of the last documented contact (telephone or in-person visit), whichever occurred first. August 2024 represents the administrative closure of the database. Multivariate Cox proportional hazards models were applied to estimate hazard ratios for cancer risk across Pb quartiles. The models were adjusted for age at blood draw (<50 or ≥50), smoking status, cancer in first-degree relatives, oophorectomy, oral contraception, and hormone replacement therapy. All statistical analyses were performed using STATISTICA software (version 13.3, TIBCO Software Inc., Palo Alto, CA, USA).

Blood Pb levels were categorized into quartiles (Q1–Q4) by sorting values in ascending order and dividing them into four equally sized groups. Quartile ranges derived from the study cohort were used for subsequent gene analyses. In each regression model, the reference category was defined as the quartile with the lowest proportion of events relative to the total number of individuals, in order to facilitate interpretability and comparability of hazard ratios across models. Due to the low frequency of events within individual quartiles, quartiles were combined when necessary to improve the stability of the estimates.

To reduce the probability of false-positive findings, only associations with HR > 4 and *p* < 0.01 are presented in the main text; all other results are provided in the Supplementary Materials.

## 5. Conclusions

In summary, variation in genes involved in lead (Pb) toxicity pathways may modify the association between blood Pb levels and cancer risk. Our findings suggest that incorporating targeted genotyping may help identify Pb exposure ranges associated with differential risk profiles.

Given the observational nature of this study and the toxicity of Pb even at low exposure levels, these results require validation in independent study populations.

Taken together, blood Pb levels combined with DNA polymorphisms in key genes may represent a potentially useful marker set for identifying individuals at increased cancer risk in the context of Pb exposure.

## Figures and Tables

**Table 1:** Characteristics of 2782 women in the cohort.

Characteristics	Unaffected Women	Women with Cancer	Mean Pb Level 14.38 µg/L, SD 7.77	Univariate Cancer Risk HR; (95% CI); *p*	Multivariate Cancer Risk HR; (95% CI); *p*
Blood Pb levels by quartiles µg/L					
Q1 <9.44	665 (23.9%)	31 (1.1%)	7.52 ± 1.34		
Q2 9.44–12.58	642 (23.1%)	53 (1.9%)	10.98 ± 0.90	HR 1.47; 95% CI (0.94–2.29); *p* = 0.089	HR 1.30; 95% CI (0.83–2.04); *p* = 0.25
Q3 12.59–17.16	631 (22.7%)	64 (2.3%)	14.74 ± 1.38	HR 1.60; 95% CI (1.04–2.47); *p* = 0.031	HR 1.32; 95% CI (0.84–2.07); *p* = 0.22
Q4 >17.16	634 (22.8%)	62 (2.2%)	24.31 ± 8.9	HR 1.50; 95% CI (0.97–2.32); *p* = 0.06	HR 1.19; 95% CI (0.75–1.89); *p* = 0.45
Genotype					
*APOB* rs1367117 GG ref	1187 (42.6%)	96 (3.5%)	14.14 ± 7.7		
*APOB* rs1367117 non-GG	1385 (49.7%)	114 (4.2%)	14.66 ± 7.85	HR 1.01; 95% CI (0.77–1.33); *p* = 0.89	HR 1.03; 95% CI (0.78–1.35); *p* = 0.82
*MKI67* rs11016073 AA ref	1551 (55.8%)	133 (4.7%)	14.37 ± 7.66		
*MKI67* rs11016073 non-AA	1021 (36.8%)	77 (2.7%)	14.41 ± 7.93	HR 1.08; 95% CI (0.82–1.44); *p* = 0.55	HR 0.92; 95% CI (0.70–1.23); *p* = 0.60
Type of cancer Family Syndrome					
HBOC or HBC	1109 (40%)	110 (4%)	14.71 ± 8.45	HR 1.54; 95% CI (1.17–2.02); *p* = 0.001	HR 1.58; 95% CI (1.17–2.12); *p* = 0.002
CFA	1545 (55%)	135 (4.8%)	14.02 ± 7.11	HR 1.42; 95% CI (1.07–1.88); *p* = 0.015	HR 1.41; 95% CI (1.01–1.96); *p* = 0.04
Not found	515 (18.5%)	27 (0.9%)	14.22 ± 7.66	HR 1.39; 95% CI (0.92–2.08); *p* = 0.11	HR 1.13; 95% CI (0.68–1.88); *p* = 0.63
Cancers in first degree relatives					
No	424 (15.2%)	34 (1.2%)	13.80 ± 7.72		
Yes	2148 (77.2%)	176 (6.4%)	14.5 ± 7.77	HR 1.05; 95% CI (0.73–1.52); *p* = 0.78	HR 0.98; 95% CI (0.68–1.43); *p* = 0.95
Oral contraceptives					
No	1874 (67.3%)	169 (6.1%)	14.95 ± 7.69		
Yes	698 (25.1%)	41 (1.5%)	12.83 ± 7.77	HR 0.83; 95% CI (0.59–1.18); *p* = 0.31	HR 1.06; 95% CI (0.74–1.53); *p* = 0.73
Oophorectomy					
No	2412 (86.7%)	193 (6.9%)	14.26 ± 7.69		
Yes	160 (5.75%)	17 (0.65%)	16.24 ± 8.60	HR 1.34; 95% CI (0.81–2.21); *p* = 0.24	HR 1.19; 95% CI (0.71–1.97); *p* = 0.50
Hormone replacement therapy					
No	2029 (72.9%)	158 (5.6%)	14.10 ± 7.56		
Yes	543 (19.6%)	52 (1.9%)	15.44 ± 8.42	HR 1.19; 95% CI (0.88–1.64); *p* = 0.24	HR 1.01; 95% CI (0.73–1.40); *p* = 0.93
Smoking status					
No	1355 (48.7%)	107 (3.8%)	13.44 ± 7.10		
Yes (former or current)	1217 (43.8%)	103 (3.7%)	15.43 ± 8.32	HR 1.02; 95% CI (0.78–1.34); *p* = 0.83	HR 0.96; 95% CI (0.73–1.27); *p* = 0.80
Age					
<50	1131 (40.7%)	55 (1.9%)	11.64 ± 6.15		
≥50	1441 (51.9%)	155 (5.6%)	16.43 ± 8.20	HR 1.74; 95% CI (1.28–2.37); *p* = 0.0004	HR 1.67; 95% CI (1.17–2.39); *p* = 0.004

**Table 2:** Incident cancers detected in the cohort.

Cancer Site	n	Cases	Mean Pb Level µg/L, ±SD
None	2572		14.23 ± 7.57
Any Cancer	210	100	16.27 ± 9.71
Breast	106	50.43	16.89 ± 11.11
Lung	10	4.7	23.41 ± 10.76
Uterus	12	5.7	17.33 ± 11.86
Leukemia	4	1.9	17.89 ± 11.27
Lymphoma	3	1.4	17.63 ± 1.59
Bladder	4	1.9	17.07 ± 5.32
Thyroid	10	4.8	16.39 ± 8.32
Ovarian	14	6.7	15.70 ± 8.62
Cervix	5	2.4	16.17 ± 4.76
Myeloma	1	0.47	15.17 ± 5.41
Melanoma	5	2.4	12.27 ± 5.13
Liver	1	0.47	14.21
Stomach	5	2.3	13.77 ± 6.73
Skin	9	4.3	13.59 ± 5.43
Glioma	1	0.47	13.50
Chondroma	1	0.47	13.35
Colon	10	4.9	11.29 ± 4.53
Partoid Gland	1	0.47	12.56
Kidney	5	2.4	12.04 ± 3.69
Abdominal Cavity	1	0.47	11.11
Pancreas	2	0.95	10.6 ± 2.81

**Table 3:** Hazard ratios for breast cancer by blood Pb level irrespective on age with *MKI67* rs11016073 non-AA genotype (quartiles).

			Univariate COX Regression			Multivariate COX Regression *		
Blood Pb Level µg/L	Cases	Unaffected	HR	95% CI	*p*	HR	95% CI	*p*
Q1 <9.44	6 (2.2%)	267 (97.8%)	1.46	0.44–4.82	0.53	1.72	0.49–5.94	0.39
Q2 9.44–12.58	19 (7.2%)	245 (92.8%)	**4.58**	**1.70–12.30**	**0.0025**	**4.80**	**1.75–13.13**	**0.0022**
Q3 12.59–17.16	10 (3.8%)	255 (96.2%)	1.95	0.67–5.73	0.22	2.24	0.76–6.64	0.14
Q4 ref >17.16	5 (2%)	254 (98%)						

* Adjusted to age, smoking, first-degree relatives, adnexectomy, oral contraception, and hormone replacement therapy. Bold values indicate statistically significant results (*p* < 0.01).

**Table 4:** Hazard ratios for breast cancer by blood Pb level for women above 50 years of age with *MKI67* rs11016073 non-AA genotype (quartiles).

			Univariate COX Regression			Multivariate COX Regression *		
Blood Pb Level µg/L	Cases	Unaffected	HR	95% CI	*p*	HR	95% CI	*p*
Q1 <9.44	4 (4.9%)	78 (95.1%)	4.06	0.90–18.25	0.067	5.90	1.29–26.98	0.022
Q2 9.44–12.58	8 (6.5%)	115 (93.5%)	5.38	1.42–20.34	0.013	**5.78**	**1.52–21.90**	**0.009**
Q3 12.59–17.16	7 (4.1%)	166 (95.9%)	2.73	0.70–10.56	0.14	3.61	0.92–14.17	0.065
Q4 ref >17.16	3 (1.5%)	200 (98.5%)						

* Adjusted to smoking, first-degree relatives, adnexectomy, oral contraception, and hormone replacement therapy. Bold values indicate statistically significant results (*p* < 0.01).

**Table 5:** Hazard ratios for breast cancer by blood Pb level for women above 50 years of age with *MKI67* rs11016073 AA genotype (quartiles).

			Univariate COX Regression			Multivariate COX Regression *		
Blood Pb Level µg/L	Cases	Unaffected	HR	95% CI	*p*	HR	95% CI	*p*
Q1 <9.44	3 (2.9%)	100 (97.1%)	1.89	0.42–8.47	0.40	1.93	0.43–8.67	0.38
Q2 ref 9.44–12.58	4 (1.9%)	213 (98.1%)						
Q3 12.59–17.16	13 (4.6%)	269 (95.4%)	2.45	0.79–7.52	0.11	2.56	0.82–7.90	0.10
Q4 >17.16	26 (8%)	300 (92%)	**4.16**	**1.45–11.94**	**0.007**	**4.26**	**1.48–12.29**	**0.007**

* Adjusted to smoking, first-degree relatives, adnexectomy, oral contraception, and hormone replacement therapy. Bold values indicate statistically significant results (*p* < 0.01).

**Table 6:** Hazard ratios for any cancer according to blood Pb levels for women below 50 years of age *APOB* rs1367117 non-GG genotype (quartiles).

			Univariate COX Regression			Multivariate COX Regression *		
Blood Pb Level µg/L	Cases	Unaffected	HR	95% CI	*p*	HR	95% CI	*p*
Q1 ref <9.44	5 (1.8%)	281 (98.2%)						
Q2 9.44–12.58	12 (6.6%)	172 (93.4%)	3.34	1.17–9.49	0.023	3.339	1.17–9.50	0.023
Q3 12.59–17.16	11 (9.4%)	107 (90.6%)	**4.56**	**1.58–13.16**	**0.005**	**4.57**	**1.57–13.27**	**0.005**
Q4 >17.16	5 (7.4%)	63 (92.6%)	3.30	0.95–11.46	0.059	3.31	0.95–11.57	0.059

* Adjusted to smoking, first-degree relatives, adnexectomy, oral contraception, and hormone replacement therapy. Bold values indicate statistically significant results (*p* < 0.01).

**Table 7:** Hazard ratios for breast cancer by blood Pb level irrespective on age genotype *APOB* non-GG and *MKI67* non-AA (quartiles).

			Univariate COX Regression			Multivariate COX Regression *		
Blood Pb Level µg/L	Cases	Unaffected	HR	95% CI	*p*	HR	95% CI	*p*
Q1 <9.44	3 (1.9%)	151 (98.1%)	3.32	0.34–32.12	0.29	3.88	0.38–38.93	0.24
Q2 9.44–12.58	12 (8.7%)	125 (91.3%)	13.86	1.79–106.96	0.011	**15.32**	**1.96–119.60**	**0.009**
Q3 12.59–17.16	7 (5%)	133 (95%)	7.17	0.88–58.39	0.06	8.53	1.03–70.33	0.046
Q4 ref >17.16	1 (0.8%)	121 (99.2%)						

* Adjusted to age, smoking, first-degree relatives, adnexectomy, oral contraception, and hormone replacement therapy. Bold values indicate statistically significant results (*p* < 0.01).

**Table 8:** Hazard ratios for breast cancer by blood Pb level for genotype *APOB* GG and *MKI67*AA irrespective on age (quartiles).

			Univariate COX Regression			Multivariate COX Regression *		
Blood Pb Level µg/L	Cases	Unaffected	HR	95% CI	*p*	HR	95% CI	*p*
Q1 ref <9.44	4 (2.3%)	168 (97.7%)						
Q2 9.44–12.58	5 (2.7%)	176 (97.3%)	1.90	0.42–8.52	0.40	2.10	0.43–10.25	0.35
Q3 12.59–17.16	3 (1.6%)	177 (98.4%)	1.64	0.39–6.90	0.49	1.80	0.41–7.74	0.42
Q4 >17.16	17 (8.8%)	176 (91.2%)	**5.21**	**1.52–17.79**	**0.008**	**5.65**	**1.59–20.03**	**0.007**
Q1–3 ref vs. Q4 <17.16 >17.16	12 (2.2%) 17 (8.9%)	521 (97.8%) 176 (91.1%)	**3.53**	**1.68–7.41**	**0.0008**	**3.71**	**1.66–8.30**	**0.001**

* Adjusted to age, smoking, first-degree relatives, adnexectomy, oral contraception, and hormone replacement therapy. Bold values indicate statistically significant results (*p* < 0.01).

**Table 9:** Hazard ratios for breast cancer by blood Pb level for genotype *APOB*GG and *MKI67*AA above 50 years of age (quartiles).

			Univariate COX Regression			Multivariate COX Regression *		
Blood Pb Level µg/L	Cases	Unaffected	HR	95% CI	*p*	HR	95% CI	*p*
Q1 <9.44	1 (2%)	48 (98%)	1.80	0.16–20.00	0.63	2.06	0.18–23.65	0.56
Q2 9.44–12.58	3 (2.9%)	99 (97.1%)	1.89	0.31–11.35	0.48	1.93	0.31–11.86	0.47
Q3 ref 12.59–17.16	2 (1.5%)	127 (98.5%)						
Q4 >17.16	15 (10%)	134 (90%)	6.65	1.51–29.09	0.011	6.64	1.46–30.13	0.014
Q1–3 ref vs. Q4 <17.16 >17.16	6 (2.1%) 15 (10%)	274 (97.9%) 134 (90%)	**4.58**	**1.77–11.82**	**0.0016**	**4.43**	**1.68–11.68**	**0.002**

* Adjusted to smoking, first-degree relatives, adnexectomy, oral contraception, and hormone replacement therapy. Bold values indicate statistically significant results (*p* < 0.01).

## Data Availability

The original contributions presented in this study are included in the article/Supplementary Materials. Further inquiries can be directed to the corresponding author.

## Supplementary Materials

The supporting information can be downloaded at: https://www.mdpi.com/article/10.3390/ijms27052317/s1.

## Abbreviations

IARC—International Agency for Research on Cancer; ICP-MS–Inductively Coupled Plasma Mass Spectrometry; CRM—certified reference material; LAMP—Pb And Multi-element Proficiency Program; CDC—Center for Disease Control; QMEQAS—Quebec Multielement External Quality Assessment Scheme; HR—hazard ratio; RR—relative risk; LOD—limit of detection; LOQ—limit of quantification; SD—standard deviation; RSD—relative standard deviation; QCS2—Quick-Change Socket; HBC—Hereditary Breast Cancer; HBOC—Hereditary Breast and Ovarian Cancer; CFA—Cancer Familial Aggregation
