# Supplementary material for: Evidence for Genotype-Specific Optimal Blood Lead Levels for Cancer Risk: MKI67 rs11016073 and APOB rs1367117 in a Female Prospective Cohort
Source: Int J Mol Sci. 2026 Mar 1;27(5):2317. doi: 10.3390/ijms27052317 (PMC12986431; doi:10.3390/ijms27052317)
Supplement: Supplementary file 1 [file ijms-27-02317-s001.zip › ijms-4106607-supplementary.pdf]

Table S.1 Other studies results

N	Type of study group	Type of Cancer	Results<br><br>Q1 or tertile 1 reference	References
EPIC-Italy 47749<br><br>NSHDS 95000	Populational	B-cell NHL and Multiple myeloma<br><br>Total cases194	Q4 (87.531-400.843 µg/L)OR 0.93;CI95%(0.43-2.02);p 0.852	[14]
The Finnish cohort 20.752<br><br>The Great Britain cohort 9122	Occupational exposure	Total cases 7000	Q1<20µg/dL<br><br>Q2 20-29 µg/dL<br><br>Q3 30-39 µg/dL<br><br>Q4 40+ µg/dL	[15]
		Brain malignant	Q4HR1.71;CI95%(0.94-3.12);p 0.04	
		Glioma	Q4HR1.4;CI95%(0.71-2.76);p 0.05	
		Meningioma	Q3HR2.16;CI95%(0.66-7.07);p 0.35	
		Oesophagus	Q3HR2.00;CI95%(1.08-3.71;p 0.009	

		Hodgkin's lymphoma	Q3HR2.37;CI95%(0.87-6.43);p 0.15	
		Lung	Q4HR1.57;CI95%(1.30-1.90); p<0.0001	
		Melanoma	Q2HR1.09;CI95%(0.8-1.48);p 0.95	
		Rectum	Q4HR1.49;CI95%(1.03-2.17);p 0.55	
		Stomach	Q2HR1.55;CI95%(1.10-2.18);p 0.84	
		Kidney	Q2HR1.05;CI95%(0.75-1.48);p 0.51	
		Bladder	Q4HR1.24;CI95%(0.87-1.75);p 0.38	
		Larynx	Q4HR1.92;CI95%(0.94-3.91);p 0.13	
NHANES 16034	Populational	Total cases 202<br>Skin cancer	Q4(0.11μg/dL)OR<br>0.71;CI95%(0.45–1.11);p 0.481	[16]
NHANES 94337	Populational	Cases788 Breast cancer	OR0.83;CI95%[0.75. 0.92]; p <0.001	[17]
		Cases 113<br>Ovarian Cancer	OR1.06;CI95%[1.00- 1.13];p 0.044	

		Cases784<br>Prostate Cancer	OR1.08;CI95%[1.04- 1.11]; p < 0.001	
		Cases 33<br>Testicular cancer	OR1.06;CI95%[0.97. 1.16]; p 0.618	
CPS-II NC 184 185	Populational	B-cell NHL and Multiple myeloma<br>Total cases 375	Q4(40.94+µg/L)RR1.52; CI95%(1.02. 2.25); p 0.08	[18]
20 741	Occupational exposure	Lung Cancer<br>Total cases 53	Q3(1.4-1.9µmol/L)OR1.7;CI95%(0.7-3.9)	[19]
4573	Populational	Total cases 541 Any cancers	Q4HR 1.51;CI95%(1.07.2.12);p 0.04	[20]
367	Populational	Total cases 100 Lung cancer	OR0.98;CI(0.81-1.19);p 0.834	[21]
1725	Populational	Total cases<br>585	Q3 fingernailslevels OR2.32;CI(1.58-3.46)	[22]

		Gastroesophageal cancers		
EPIC-Italy 300	Populational	Total cases 150 Breast cancer	Tertile2(1.5-2.3μg/L) OR0.74;CI(0.23-2.34);p0.603	[23]
EPIC-Spain 600	Populational	Total cases 300 Breast cancer	Q3(0.3-0.5ng/ml) OR1.3;CI(0.95-1.77);p 0.1	[24]
NHANES 40486	Populational	Total cases 67 Bladder cancer	OR 2.946 ;CI1.025 to 8.465, P 0.047)	[25]
NHANES 13 467	Populational	Total cases 9 esophagus cancer	OR 15.21;CI 3.08 to 74.97, P 0.001)	[26]
		Total cases 11 gastric cancer	OR 6.09;CI 1.99 to 18.62, P 0.002)	
2927	Populational	Total cases 116 Breast cancer	HR 2.64;CI 1.21 to 5.76, P 0.014	[27]
		Total cases 239 Any cancer		

Table S.2 Hazard ratios for any cancer by blood Pb level for whole group (quartiles).

			Univariate COX Regression			Multivariate COX Regression *		
**Blood Pb level** μg/L	**Cases**	**Unaffected**	**HR**	**95% CI**	***p*-value**	**HR**	**95% CI**	***p*-value**
Q1 ref <9.44	31(4.4%)	665 (95.6%)	—	—	—	—	—	—
Q2 9.44-12.58	53(7.6%)	642(92.4%)	1.46	0.94-2.29	0.089	1.30	0.83- 2.05	0.24
Q3 12.59-17.16	64(9.2%)	631(90.8%)	1.60	1.04-2.46	0.03	1.319	0.84- 2.06	0.22
Q4 >17.16	62(8.9%)	634(91.1%)	1.50	0.97-2.32	0.06	1.19	0.75- 1.89	0.45

***Adjusted to (age, smoking, 1stdegree relatives, adnexectomy, oral contraception, hormone replacement therapy)**

Table S.3 Hazard ratios for any cancer on whole group with MKI67 rs11016073 nonAA genotype (quartiles).

			Univariate COX Regression			Multivariate COX Regression *		
**Blood Pb level** μg/L	**Cases**	**Unaffected**	**HR**	**95% CI**	***p*-value**	**HR**	**95% CI**	***p*-value**
Q1 <9.44	15(6.4%)	267(94.6%)	1.17	0.57-2.38	0.65	1.39	0.65-2.93	0.38
Q2 9.44-12.58	25(9.3%)	245(90.7%)	1.94	1.034-3.64	0.039	2.14	1.12-4.07	0.02
Q3 12.59-17.16	21(7.7%)	255(92.3%)	1.28	0.67-2.46	0.45	1.36	0.70-2.63	0.35
Q4 ref >17.16	16(6%)	254(94%)	—	—	—	—	—	—

***Adjusted to (age, smoking, 1stdegree relatives, adnexectomy, oral contraception, hormone replacement therapy)**

Table S.4 Hazard ratios for any cancer on whole group with MKI rs11016073 AA genotype (quartiles).

			Univariate COX Regression			Multivariate COX Regression *		
**Blood Pb level** μg/L	**Cases**	**Unaffected**	**HR**	**95% CI**	***p*-value**	**HR**	**95% CI**	***p*-value**
Q1 ref <9.44	16(3.9%)	398(96.1%)	—	—	—	—	—	—
Q2 9.44-12.58	28(6.6%)	397(93.4%)	1.36	0.74-2.53	0.31	1.15	0.61-2.17	0.64
Q3 12.59-17.16	43(10.3%)	376(89.7%)	2.07	1.16-3.68	0.0129	1.58	0.86-2.89	0.13
Q4 >17.16	46(10.8%)	380(89.2%)	2.068	1.16-3.66	0.0126	1.56	0.84-2.87	0.15

***Adjusted to (age, smoking, 1st degree relatives, adnexectomy, oral contraception, hormone replacement therapy)**

Table S.5 Hazard ratios for any cancer on whole group with APOB rs1367117 nonGG genotype (quartiles).

			Univariate COX Regression			Multivariate COX Regression *		
**Blood Pb level** μg/L	**Cases**	**Unaffected**	**HR**	**95% CI**	***p*-value**	**HR**	**95% CI**	***p*-value**
Q1 ref <9.44	17(4.3%)	382(95.7%)	—	—	—	—	—	—
Q2 9.44-12.58	28(7.5%)	346(92.5%)	1.57	0.86-2.87	0.14	1.45	0.78-2.67	0.23
Q3 12.59-17.16	41(11%)	332(89%)	2.13	1.21-3.76	0.008	1.88	1.04-3.39	0.036
Q4 >17.16	28(8%)	325(92%)	1.40	0.76-2.57	0.26	1.19	0.62-2.26	0.59

***Adjusted to (age, smoking, 1st degree relatives, adnexectomy, oral contraception, hormone replacement therapy)**

Table S.6 Hazard ratios for any cancer by blood Pb level for whole group APOB rs1367117 GG genotype (quartiles).

			Univariate COX Regression			Multivariate COX Regression *		
**Blood Pb level** µg/L	**Cases**	**Unaffected**	**HR**	**95% CI**	***p*-value**	**HR**	**95% CI**	***p*-value**
Q1 <9.44	14(4.7%)	283(95.3%)	—	—	—	1.20	0.60-2.41	0.59
Q2 9.44-12.58	25(7.8%)	296(92.2%)	1.339	0.69-2.58	0.38	1.38	0.77-2.46	0.27
Q3 ref 12.59-17.16	23(6.7%)	322(93.3%)	1.089	0.55-2.12	0.80	—	—	—
Q4 >17.16	34(9.1%)	343(90.9%)	1.56	0.83-2.92	0.16	1.38	0.80-2.37	0.23

***Adjusted to (age, smoking, 1st degree relatives, adnexectomy, oral contraception, hormone replacement therapy)**

Table S.7 Hazard ratios for breast cancer by blood Pb level irrespective on age (quartiles).

			Univariate COX Regression			Multivariate COX Regression *		
**Blood Pb level** µg/L	**Cases**	**Unaffected**	**HR**	**95% CI**	***p*-value**	**HR**	**95% CI**	***p*-value**
Q1 ref <9.44	15(2.3%)	665(97.7%)	—	—	—	—	—	—
Q2 9.44-12.58	28(4.2%)	642(95.8%)	1.62	0.86-3.04	0.13	1.60	0.84- 3.027	0.14
Q3 12.59-17.16	28(4.3%)	631(95.7%)	1.48	0.79-2.78	0.21	1.46	0.75- 2.80	0.25
Q4 >17.16	35(5.3%)	634(94.7%)	1.78	0.97-3.28	0.06	1.68	0.88- 3.23	0.11

***Adjusted to (age, smoking, 1st degree relatives, adnexectomy, oral contraception, hormone replacement therapy)**

Table S.8 Hazard ratios for breast cancer by blood Pb level irrespective on age with MKI rs11016073 AA genotype (quartiles).

			Univariate COX Regression			Multivariate COX Regression *		
**Blood Pb level** μg/L	**Cases**	**Unaffected**	**HR**	**95% CI**	***p*-value**	**HR**	**95% CI**	***p*-value**
Q1 <9.44	9(2.3%)	398(97.7%)	1.27	0.50-3.21	0.61	1.28	0.49- 3.28	0.60
Q2 ref 9.44-12.58	9(2.4%)	397(97.6%)	—	—	—	—	—	—
Q3 12.59-17.16	18(4.6%)	376(95.4%)	1.99	0.89-4.43	0.09	1.97	0.87- 4.45	0.10
Q4 >17.16	30(7.4%)	380(92.6%)	3.06	1.45-6.45	0.003	3.09	1.44- 6.63	0.0037

***Adjusted to (age, smoking, 1stdegree relatives, adnexectomy, oral contraception, hormone replacement therapy)**

Table S.9 Hazard ratios for breast cancer by blood Pb level irrespective on age APOB rs1367117 nonGG genotype (quartiles).

			Univariate COX Regression			Multivariate COX Regression *		
**Blood Pb level** μg/L	**Cases**	**Unaffected**	**HR**	**95% CI**	***p*-value**	**HR**	**95% CI**	***p*-value**
Q1 <9.44	7(1.8%)	382(98.2%)	—	—	—	—	—	—
Q2 9.44-12.58	16(4.5%)	346(95.5%)	2.18	0.89-5.30	0.085	2.20	0.89- 5.40	0.08
Q3 ref 12.59-17.16	22(6.3%)	332(93.7%)	2.80	1.19-6.57	0.017	2.81	1.16- 6.80	0.02
Q4 >17.16	14(4.2%)	325(95.8%)	1.70	0.68-4.24	0.25	1.67	0.64- 4.39	0.29

***Adjusted to (age, smoking, 1stdegree relatives, adnexectomy, oral contraception, hormone replacement therapy)**

Table S.10 Hazard ratios for breast cancer by blood Pb level irrespective on age APOB rs1367117 GG genotype (quartiles).

			Univariate COX Regression			Multivariate COX Regression *		
**Blood Pb level** μg/L	**Cases**	**Unaffected**	**HR**	**95% CI**	***p*-value**	**HR**	**95% CI**	***p*-value**
Q1 <9.44	8(2.8%)	283(97.2%)	1.92	0.66-5.56	0.22	2.02	0.67-6.098	0.21
Q2 9.44-12.58	12(3.9%)	296(96.1%)	2.23	0.83-5.95	0.10	2.30	0.84-6.23	0.10
Q3 ref 12.59-17.16	6(2%)	299(98%)	—	—	—	—	—	—
Q4 >17.16	21(6.4%)	309(93.6%)	3.36	1.35-8.33	0.008	3.22	1.28-8.092	0.012

***Adjusted to (age, smoking, 1st degree relatives, adnexectomy, oral contraception, hormone replacement therapy)**

Table S.11 Hazard ratios for any cancer by blood Pb level for women above 50 years of age (quartiles).

			Univariate COX Regression			Multivariate COX Regression *		
**Blood Pb level** μg/L	**Cases**	**Unaffected**	**HR**	**95% CI**	***p*-value**	**HR**	**95% CI**	***p*-value**
Q1 <9.44	19(9.7%)	178(90.3%)	1.23	0.71-2.04	0.47	1.23	0.72-2.08	0.44
Q2 9.44-12.58	34(9.4%)	328(90.6%)	1.06	0.69-1.63	0.78	1.06	0.69- 1.64	0.76
Q3 12.59-17.16	48(9.4%)	435(90.6%)	1.05	0.70-1.53	0.84	1.05	0.70- 1.56	0.80
Q4 ref >17.16	54(9.8%)	500(90.2%)	—	—	—	—	—	—

***Adjusted to (smoking, 1st degree relatives, adnexectomy, oral contraception, hormone replacement therapy)**

Table S.12 Hazard ratios for any cancer on women above 50 years of age with MKI rs11016073 nonAA genotype (quartiles).

			Univariate COX Regression			Multivariate COX Regression *		
**Blood Pb level** µg/L	**Cases**	**Unaffected**	**HR**	**95% CI**	***p*-value**	**HR**	**95% CI**	***p*-value**
Q1 <9.44	9(10.4%)	78(89.6%)	1.92	0.82-4.45	0.12	2.03	0.86-4.76	0.10
Q2 9.44-12.58	13(10.2%)	115(89.8%)	1.83	0.86-3.92	0.11	1.89	0.88-4.04	0.099
Q3 12.59-17.16	15(8.3%)	166(91.7%)	1.26	0.61-2.63	0.52	1.32	0.63-2.77	0.45
Q4 ref >17.16	14(6.5%)	200(93.5%)	—	—	—	—	—	—

***Adjusted to (smoking, 1st degree relatives, adnexectomy, oral contraception, hormone replacement therapy)**

Table S.13 Hazard ratios for any cancer on women above 50 years of age with MKI rs11016073 AA genotype (quartiles).

			Univariate COX Regression			Multivariate COX Regression *		
**Blood Pb level** µg/L	**Cases**	**Unaffected**	**HR**	**95% CI**	***p*-value**	**HR**	**95% CI**	***p*-value**
Q1 <9.44	10(9.1%)	100(90.9%)	1.19	0.56-2.54	0.64	1.19	0.56-2.54	0.64
Q2 ref 9.44-12.58	21(9%)	213(91%)	—	—	—	—	—	—
Q3 12.59-17.16	33(11%)	269(89%)	1.18	0.68-2.05	0.53	1.19	0.68-2.07	0.54
Q4 >17.16	40(11.8%)	300(88.2%)	1.23	0.72-2.09	0.43	1.23	0.72-2.10	0.44

***Adjusted to (smoking, 1st degree relatives, adnexectomy, oral contraception, hormone replacement therapy)**

Table S.14 Hazard ratios for any cancer by blood Pb level on women above 50 years of age APOB rs1367117 nonGG genotype (quartiles).

			Univariate COX Regression			Multivariate COX Regression *		
**Blood Pb level** µg/L	**Cases**	**Unaffected**	**HR**	**95% CI**	***p*-value**	**HR**	**95% CI**	***p*-value**
Q1 <9.44	12(10.7%)	101(89.3%)	1.56	0.77-3.13	0.21	1.51	0.74-3.08	0.25
Q2 9.44-12.58	16(8.5%)	174(91.5%)	1.18	0.62-2.24	0.60	1.15	0.60-2.18	0.67
Q3 12.59-17.16	30(11.8%)	225(88.2%)	1.56	0.91-2.69	0.10	1.58	0.91-2.74	0.10
Q4 ref >17.16	23(8.1%)	262(91.9%)	—	—	—	—	—	—

***Adjusted to (smoking, 1st degree relatives, adnexectomy, oral contraception, hormone replacement therapy)**

Table S.15 Hazard ratios for any cancer by blood Pb level on women above 50 years of age APOB rs1367117 GG genotype (quartiles).

			Univariate COX Regression			Multivariate COX Regression *		
**Blood Pb level** µg/L	**Cases**	**Unaffected**	**HR**	**95% CI**	***p*-value**	**HR**	**95% CI**	***p*-value**
Q1 <9.44	7(8.4%)	77(91.6%)	1.39	0.58-3.349	0.45	1.35	0.56-3.29	0.49
Q2 9.44-12.58	18(10.5%)	154(89.5%)	1.44	0.75-2.77	0.27	1.45	0.74-2.82	0.27
Q3 ref 12.59-17.16	18(7.9%)	210(92.1%)	—	—	—	—	—	—
Q4 >17.16	31(11.6%)	238(88.4%)	1.50	0.84-2.69	0.16	1.47	0.81-2.66	0.20

***Adjusted to (smoking, 1st degree relatives, adnexectomy, oral contraception, hormone replacement therapy)**

Table S.16 Hazard ratios for breast cancer by blood Pb level for women above 50 years of age (quartiles).

			Univariate COX Regression			Multivariate COX Regression *		
**Blood Pb level** µg/L	**Cases**	**Unaffected**	**HR**	**95% CI**	***p*-value**	**HR**	**95% CI**	***p*-value**
Q1 <9.44	7(3.8%)	178(96.2%)	1.18	0.46-3.006	0.72	1.27	0.50- 3.24	0.61
Q2 ref 9.44-12.58	12(3.6%)	328(96.4%)	—	—	—	—	—	—
Q3 12.59-17.16	20(4.4%)	435(95.6%)	1.15	0.56-2.35	0.70	1.19	0.57- 2.44	0.63
Q4 >17.16	29(5.5%)	500(94.5%)	1.41	0.72-2.77	0.31	1.36	0.69- 2.67	0.37

***Adjusted to (smoking, 1st degree relatives, adnexectomy, oral contraception, hormone replacement therapy**

Table S.17 Hazard ratios for breast cancer by blood Pb level for women above 50 years of age APOB rs1367117 nonGG genotype (quartiles).

			Univariate COX Regression			Multivariate COX Regression *		
**Blood Pb level** µg/L	**Cases**	**Unaffected**	**HR**	**95% CI**	***p*-value**	**HR**	**95% CI**	***p*-value**
Q1 <9.44	4(3.8%)	101(96.2%)	1.09	0.35-3.45	0.87	1.12	0.35-3.59	0.84
Q2 9.44-12.58	7(3.9%)	174((96.1%)	1.094	0.42-2.82	0.85	1.11	0.42-2.89	0.82
Q3 12.59-17.16	15(6.25%)	225(93.75%)	1.64	0.75-3.58	0.21	1.72	0.78-3.78	0.17
Q4 ref >17.16	11(4%)	262(96%)	—	—	—	—	—	—

***Adjusted to (smoking, 1st degree relatives, adnexectomy, oral contraception, hormone replacement, hormone replacement therapy)**

Table S.18 Hazard ratios for breast cancer by blood Pb level on women above 50 years of age APOB rs1367117 GG genotype (quartiles).

			Univariate COX Regression			Multivariate COX Regression *		
**Blood Pb level** µg/L	**Cases**	**Unaffected**	**HR**	**95% CI**	***p*-value**	**HR**	**95% CI**	***p*-value**
Q1 <9.44	3(3.8%)	77(96.2%)	2.13	0.50-8.95	0.30	2.26	0.53-9.65	0.26
Q2 9.44-12.58	5(3.2%)	154(96.8%)	1.47	0.42-5.08	0.54	1.43	0.40-5.013	0.57
Q3 ref 12.59-17.16	5(2.4%)	210(97.6%)	—	—	—	—	—	—
Q4 >17.16	18(7.1%)	238(92.9%)	3.12	1.15-8.40	0.024	2.83	1.03-7.74	0.042

***Adjusted to (smoking, 1st degree relatives, adnexectomy, oral contraception, hormone replacement therapy)**

Table S.19 Hazard ratios for any cancer by blood Pb level for women below 50 years of age (quartiles).

			Univariate COX Regression			Multivariate COX Regression *		
**Blood Pb level** µg/L	**Cases**	**Unaffected**	**HR**	**95% CI**	***p*-value**	**HR**	**95% CI**	***p*-value**
Q1 ref <9.44	12(2.5%)	487(97.5%)	—	—	—	—	—	—
Q2 9.44-12.58	19(5.8%)	314(94.2%)	2.07	1.003-4.27	0.089	0.048	1.01- 4.32	0.046
Q3 12.59-17.16	16(7.6%)	196 (92.4%)	2.54	1.20-5.39	0.03	0.014	1.21- 5.50	0.013
Q4 >17.16	8(5.7%)	134 (94.3%)	1.89	0.77-4.64	0.06	0.16	0.77- 4.76	0.15
Q1 <9.44 vs Q2-Q4 >9.44	12(2.5%) 43(6.3%)	487(97.5%) 644(93.7%)	2.18	1.14-4.15	0.017	2.22	1.16- 4.24	0.015

***Adjusted to (smoking, 1st degree relatives, adnexectomy, oral contraception, hormone replacement therapy)**

Table S.20 Hazard ratios for any cancer by blood Pb level for women below 50 years of age with MKI rs11016073 nonAA genotype (quartiles).

			Univariate COX Regression			Multivariate COX Regression *		
**Blood Pb level** μg/L	**Cases**	**Unaffected**	**HR**	**95% CI**	***p*-value**	**HR**	**95% CI**	***p*-value**
Q1 ref <9.44	6(2%)	298(98%)	—	—	—	—	—	—
Q2 9.44-12.58	7(3.7%)	184(96.3%)	2.68	1.005-7.16	0.048	1.05	0.21-5.33	0.94
Q3 12.59-17.16	10(8.6%)	107(91.4%)	1.61	0.52-5.04	0.40	2.76	0.61-12.5	0.18
Q4 >17.16	6(7%)	80(93%)	1.005	0.20-4.99	0.99	1.63	0.32-8.21	0.54

***Adjusted to (smoking, 1st degree relatives, adnexectomy, oral contraception, hormone replacement therapy**

Table S.21 Hazard ratios for any cancer by blood Pb level for women below 50 years of age with MKI rs11016073 AA genotype (quartiles).

			Univariate COX Regression			Multivariate COX Regression *		
**Blood Pb level** μg/L	**Cases**	**Unaffected**	**HR**	**95% CI**	***p*-value**	**HR**	**95% CI**	***p*-value**
Q1 ref <9.44	6(2%)	298(98%)	—	—	—	—	—	—
Q2 9.44-12.58	7(3.7%)	184(96.3%)	1.51	0.50-4.51	0.46	1.59	0.53-4.78	0.40
Q3 12.59-17.16	10(8.6%)	107(91.4%)	3.55	1.28-9.81	0.014	3.59	1.29-9.98	0.014
Q4 >17.16	6(7%)	80(93%)	2.65	0.84-8.29	0.09	2.98	0.94-9.48	0.06

***Adjusted to (smoking, 1st degree relatives, adnexectomy, oral contraception, hormone replacement therapy**

Table S.22 Hazard ratios for any cancer by blood Pb level for women below 50 years of age APOB rs1367117 GG genotype (quartiles).

			Univariate COX Regression			Multivariate COX Regression *		
**Blood Pb level** µg/L	**Cases**	**Unaffected**	**HR**	**95% CI**	***p*-value**	**HR**	**95% CI**	***p*-value**
Q1 ref <9.44	7(3.3%)	206(96.7%)	—	—	—	—	—	—
Q2 9.44-12.58	7(4.7%)	142(95.3%)	1.20	0.42-3.46	0.72	1.33	0.45-3.91	0.59
Q3 12.59-17.16	5(5.3%)	89(94.7%)	1.24	0.39-3.94	0.71	1.35	0.42-4.32	0.61
Q4 >17.16	3(4%)	71(96%)	1.01	0.26-3.96	0.98	1.38	0.30-4.80	0.23

***Adjusted to (smoking, 1st degree relatives, adnexectomy, oral contraception, hormone replacement therapy)**

Table S.23 Hazard ratios for breast cancer by blood Pb level for women below 50 years of age (quartiles).

			Univariate COX Regression			Multivariate COX Regression *		
**Blood Pb level** µg/L	**Cases**	**Unaffected**	**HR**	**95% CI**	***p*-value**	**HR**	**95% CI**	***p*-value**
Q1 ref <9.44	8(1.7%)	487(98.3%)	—	—	—	—	—	—
Q2 9.44-12.58	16(4.9%)	314(95.1%)	2.60	1.11-6.10	0.027	2.65	1.13- 6.24	0.025
Q3 12.59-17.16	8(4%)	196(96%)	1.96	0.73-5.24	0.18	1.93	0.72- 5.18	0.19
Q4 >17.16	6(4.3%)	134(95.7%)	2.11	0.73-6.12	0.16	2.02	0.69- 5.90	0.19
Q1 <9.44 vs<br>Q2-Q4 >9.44	8(1.7%)<br>30(4.5%)	487(98.3%)<br>644(95.5%)	2.29	1.05-5.03	0.037	2.30	1.05-5.07	0.037

***Adjusted to (smoking, 1st degree relatives, adnexectomy, oral contraception, hormone replacement therapy)**

Table S.24 Hazard ratios for breast cancer by blood Pb level for women below 50 years of age with MKI rs11016073 nonAA genotype (quartiles).

			Univariate COX Regression			Multivariate COX Regression *		
**Blood Pb level** µg/L	**Cases**	**Unaffected**	**HR**	**95% CI**	***p*-value**	**HR**	**95% CI**	***p*-value**
Q1 ref <9.44	2(1.1%)	189(98.9%)	—	—	—	—	—	—
Q2 9.44-12.58	11(7.8%)	130(92.2%)	7.35	1.62-33.23	0.009	6.86	0.61-12.5	0.012
Q3 12.59-17.16	3(3.3%)	89(96.7%)	2.65	0.44-15.92	0.28	2.23	0.36- 13.69	0.38
Q4 >17.16	2(3.6%)	54(96.4%)	3.09	0.43-22.03	0.26	2.49	0.34- 18.07	0.36
Q1 <9.44 vs Q2-Q4 >9.44	2(1.1%) 16(5.6%)	189(98.9%) 273(94.4%)	4.90	1.12-21.39	0.034	4.45	1.01- 19.58	0.04

***Adjusted to (smoking, 1st degree relatives, adnexectomy, oral contraception, hormone replacement therapy)**

Table S.25 Hazard ratios for breast cancer by blood Pb level for women below 50 years of age with MKI rs11016073 AA genotype (quartiles).

			Univariate COX Regression			Multivariate COX Regression *		
**Blood Pb level** µg/L	**Cases**	**Unaffected**	**HR**	**95% CI**	***p*-value**	**HR**	**95% CI**	***p*-value**
Q1 ref <9.44	6(1.9%)	298(98.1%)	—	—	—	—	—	—
Q2 9.44-12.58	5(2.6%)	184(97.4%)	1.017	0.30-3.35	0.97	1.13	0.34-3.78	0.012
Q3 12.59-17.16	5(4.4%)	107(95.6%)	1.72	0.52-5.68	0.36	1.76	0.52-5.87	0.38
Q4 >17.16	4(4.7%)	80(95.3%)	1.63	0.45-5.86	0.45	1.74	0.47-6.34	0.36

***Adjusted to (smoking, 1st degree relatives, adnexectomy, oral contraception, hormone replacement therapy)**

Table S.26 Hazard ratios for breast cancer by blood Pb level for women below 50 years of age APOB rs1367117 nonGG genotype (quartiles).

			Univariate COX Regression			Multivariate COX Regression *		
**Blood Pb level** µg/L	**Cases**	**Unaffected**	**HR**	**95% CI**	***p*-value**	**HR**	**95% CI**	***p*-value**
Q1 ref <9.44	3(1.1%)	281(98.9%)	—	—	—	—	—	—
Q2 9.44-12.58	9(5%)	172(95%)	4.09	1.10- 15.15	0.034	3.97	1.07- 14.75	0.039
Q3 12.59-17.16	7(6.2%)	107(93.8%)	4.91	1.26- 19.04	0.021	4.42	1.13- 17.31	0.032
Q4 >17.16	3(4.55%)	63(95.45%)	3.22	0.64- 16.04	0.15	2.95	0.58- 14.77	0.18
Q1 <9.44 vs Q2-Q4 >9.44	3(1.1%) 19(5.3%)	281(98.9%) 342(94.7%)	4.17	1.23- 14.15	0.02	3.85	1.13- 13.13	0.03

***Adjusted to (smoking, 1st degree relatives, adnexectomy, oral contraception, hormone replacement therapy)**

Table S.27 Hazard ratios for breast cancer by blood Pb level for women below 50 years of age APOB rs1367117 GG genotype (quartiles).

			Univariate COX Regression			Multivariate COX Regression *		
**Blood Pb level** µg/L	**Cases**	**Unaffected**	**HR**	**95% CI**	***p*-value**	**HR**	**95% CI**	***p*-value**
Q1 <9.44	5(2.4%)	206(97.6%)	2.67	0.31- 23.05	0.37	2.40	0.27- 20.89	0.42
Q2 9.44-12.58	7(4.7%)	142(95.3%)	4.61	0.56- 37.50	0.15	4.66	0.57- 38.16	0.15
Q3 ref 12.59-17.16	1(1.2%)	89(98.8%)	—	—	—	—	—	—
Q4 >17.16	3(4.1%)	71(95.9%)	3.80	0.39- 36.64	0.24	4.06	0.41- 39.52	0.22

***Adjusted to (smoking, 1st degree relatives, adnexectomy, oral contraception, hormone replacement therapy)**

Table S.28 Hazard ratios for any cancer by blood Pb level on whole group genotype APOB nonGG or MKI nonAA (quartiles).

			Univariate COX Regression			Multivariate COX Regression *		
**Blood Pb level** µg/L	**Cases**	**Unaffected**	**HR**	**95% CI**	***p*-value**	**HR**	**95% CI**	***p*-value**
Q1 ref <9.44	26(4.9%)	497(95.1%)	—	—	—	—	—	—
Q2 9.44-12.58	38(7.5%)	466(92.5%)	1.36	0.83-2.25	0.21	1.24	0.75-2.07	0.39
Q3 12.59-17.16	48(9.5%)	454(90.5%)	1.50	0.93-2.42	0.09	1.28	0.77-2.11	0.32
Q4 >17.16	38(7.6%)	458(92.4%)	1.17	0.70-1.93	0.53	0.96	0.56-1.64	0.88

***Adjusted to (age, smoking, 1st degree relatives, adnexectomy, oral contraception, hormone replacement therapy)**

Table S.29 Hazard ratios for any cancer by blood Pb level on whole group genotype APOB nonGG and MKI nonAA (quartiles).

			Univariate COX Regression			Multivariate COX Regression *		
**Blood Pb level** µg/L	**Cases**	**Unaffected**	**HR**	**95% CI**	***p*-value**	**HR**	**95% CI**	***p*-value**
Q1 <9.44	6(3.8%)	152(96.2%)	1.05	0.34-3.30	0.92	1.16	0.35-3.78	0.80
Q2 9.44-12.58	15(10.7%)	125(89.3%)	2.89	1.12-7.47	0.028	3.08	1.17-8.13	0.022
Q3 12.59-17.16	14(9.5%)	133(90.5%)	2.29	0.88-5.98	0.089	2.47	0.93-6.52	0.06
Q4 ref >17.16	6(4.7%)	121(95.3%)	—	—	—	—	—	—

***Adjusted to (age, smoking, 1st degree relatives, adnexectomy, oral contraception, hormone replacement therapy)**

Table S.30 Hazard ratios for any cancer by blood Pb level on whole group genotype APOB GG or MKI AA (quartiles).

			Univariate COX Regression			Multivariate COX Regression *		
**Blood Pb level** µg/L	**Cases**	**Unaffected**	**HR**	**95% CI**	***p*-value**	**HR**	**95% CI**	***p*-value**
Q1 ref <9.44	25(4.6%)	513(95.4%)	—	—	—	—	—	—
Q2 9.44-12.58	38(6.8%)	517(93.2%)	1.22	0.73-2.027	0.43	1.05	0.63-1.76	0.84
Q3 12.59-17.16	50(9.1%)	498((90.9%)	1.47	0.90-2.38	0.11	1.13	0.68-1.87	0.63
Q4 >17.16	56(9.8%)	513(90.2%)	1.58	0.98-2.54	0.057	1.18	0.71-1.97	0.50

***Adjusted to (age, smoking, 1st degree relatives, adnexectomy, oral contraception, hormone replacement therapy)**

Table S.31 Hazard ratios for any cancer by blood Pb level on whole group genotype APOB GG and MKI AA (quartiles).

			Univariate COX Regression			Multivariate COX Regression *		
**Blood Pb level** µg/L	**Cases**	**Unaffected**	**HR**	**95% CI**	***p*-value**	**HR**	**95% CI**	***p*-value**
Q1 ref <9.44	5(2.9%)	168(97.1%)	—	—	—	—	—	—
Q2 9.44-12.58	15(7.8%)	176(92.2%)	2.01	0.73-5.55	0.17	1.61	0.57-4.58	0.36
Q3 12.59-17.16	16(8.3%)	177(91.7%)	2.18	0.79-5.97	0.12	1.59	0.56-4.56	0.38
Q4 >17.16	24(12%)	176(88%)	3.04	1.15-8.01	0.024	2.21	0.79-6.15	0.12

***Adjusted to (age, smoking, 1st degree relatives, adnexectomy, oral contraception, hormone replacement therapy)**

Table S.32 Hazard ratios for any cancer by blood Pb level on whole group genotype MKI AA or APOB nonGG (quartiles).

			Univariate COX Regression			Multivariate COX Regression *		
**Blood Pb level** µg/L	**Cases**	**Unaffected**	**HR**	**95% CI**	***p*-value**	**HR**	**95% CI**	***p*-value**
Q1 ref <9.44	22(3.8%)	550(96.2%)	—	—	—	—	—	—
Q2 9.44-12.58	43(7.6%)	522(92.4%)	1.67	0.99-2.79	0.05	1.48	0.87-2.50	0.14
Q3 12.59-17.16	57(10%)	509(90%)	2.11	1.29-3.46	0.003	1.75	1.05-2.93	0.032
Q4 >17.16	52(9.4%)	501(90.6%)	1.83	1.11-3.03	0.017	1.46	0.86-2.50	0.15

***Adjusted to (age, smoking, 1st degree relatives, adnexectomy, oral contraception, hormone replacement therapy)**

Table S.33 Hazard ratios for any cancer by blood Pb level on whole group genotype MKI AA and APOB nonGG (quartiles).

			Univariate COX Regression			Multivariate COX Regression *		
**Blood Pb level** µg/L	**Cases**	**Unaffected**	**HR**	**95% CI**	***p*-value**	**HR**	**95% CI**	***p*-value**
Q1 <9.44	11(4.5%)	230(95.5%)	—	—	—	1.11	0.49-2.52	0.79
Q2 9.44-12.58	13(5.5%)	221(94.5%)	1.026	0.46-2.29	0.94	—	—	—
Q3 12.59-17.16	27(11.9%)	199(88.1%)	2.06	1.022-4.17	0.043	1.86	0.95-3.65	0.07
Q4 >17.16	22(9.7%)	204(90.3%)	1.57	0.76-3.27	0.22	1.42	0.70-2.86	0.32

***Adjusted to (age, smoking, 1st degree relatives, adnexectomy, oral contraception, hormone replacement therapy)**

Table S.34 Hazard ratios for any cancer by blood Pb level on whole group genotype MKI nonAA or APOB GG (quartiles).

			Univariate COX Regression			Multivariate COX Regression *		
**Blood Pb level** µg/L	**Cases**	**Unaffected**	**HR**	**95% CI**	***p*-value**	**HR**	**95% CI**	***p*-value**
Q1 ref <9.44	20(4.4%)	435(95.6%)	—	—	—	—	—	—
Q2 9.44-12.58	40(8.6%)	421(91.4%)	1.69	0.99-2.90	0.05	1.51	0.87-2.61	0.13
Q3 12.59-17.16	37(7.8%)	432(92.2%)	1.37	0.79-2.36	0.25	1.15	0.65-2.03	0.61
Q4 >17.16	40(8.5%)	430(91.5%)	1.46	0.85-2.50	0.16	1.16	0.65-2.057	0.60

***Adjusted to (age, smoking, 1st degree relatives, adnexectomy, oral contraception, hormone replacement therapy)**

Table S.35 Hazard ratios for any cancer by blood Pb level on whole group genotype MKI nonAA and APOB GG (quartiles).

			Univariate COX Regression			Multivariate COX Regression *		
**Blood Pb level** µg/L	**Cases**	**Unaffected**	**HR**	**95% CI**	***p*-value**	**HR**	**95% CI**	***p*-value**
Q1 <9.44	9(7.2%)	115(92.8%)	1.97	0.72-5.36	0.18	2.51	0.89-7.08	0.08
Q2 9.44-12.58	10(7.7%)	120(92.3%)	1.92	0.72-5.08	0.18	2.19	0.81-5.94	0.12
Q3 ref 12.59-17.16	7(5.4%)	122(94.6%)	—	—	—	—	—	—
Q4 >17.16	10(6.9%)	133(93.1%)	1.44	0.55-3.79	0.45	1.40	0.52-3.72	0.49

***Adjusted to (age, smoking, 1st degree relatives, adnexectomy, oral contraception, hormone replacement therapy)**

Table S.36 Hazard ratios for breast cancer by blood Pb level irrespective on age genotype APOB nonGG or MKI nonAA (quartiles).

			Univariate COX Regression			Multivariate COX Regression *		
**Blood Pb level** µg/L	**Cases**	**Unaffected**	**HR**	**95% CI**	***p*-value**	**HR**	**95% CI**	***p*-value**
Q1 ref <9.44	13(2.4%)	513(97.6%)	—	—	—	—	—	—
Q2 9.44-12.58	16(3%)	517(97%)	1.79	0.89-3.61	0.09	1.77	0.87-3.60	0.11
Q3 12.59-17.16	21(4%)	498(96%)	1.71	0.86-3.42	0.12	1.72	0.84-3.53	0.14
Q4 >17.16	24(4.4%)	513(95.6%)	1.20	0.58-2.51	0.24	1.19	0.54-2.60	0.66

***Adjusted to (age, smoking, 1st degree relatives, adnexectomy, oral contraception, hormone replacement therapy)**

Table S.37 Hazard ratios for breast cancer by blood Pb level irrespective on age genotype APOB GG or MKI AA (quartiles).

			Univariate COX Regression			Multivariate COX Regression *		
**Blood Pb level** µg/L	**Cases**	**Unaffected**	**HR**	**95% CI**	***p*-value**	**HR**	**95% CI**	***p*-value**
Q1 <9.44	12(2.4%)	496(97.6%)	0.99	0.48-2.07	0.99	1.005	0.47-2.12	0.98
Q2 ref 9.44-12.58	23(4.7%)	466(95.3%)	—	—	—	—	—	—
Q3 12.59-17.16	25(5.2%)	454(94.8%)	1.22	0.63-2.33	0.54	1.20	0.61-2.33	0.58
Q4 >17.16	18(3.8%)	458(96.2%)	1.87	1.03-3.39	0.038	1.85	1.002-3.41	0.049

***Adjusted to (age, smoking, 1st degree relatives, adnexectomy, oral contraception, hormone replacement therapy)**

Table S.38 Hazard ratios for breast cancer by blood Pb level irrespective on age genotype MKI AA or APOB nonGG (quartiles).

			Univariate COX Regression			Multivariate COX Regression *		
**Blood Pb level** μg/L	**Cases**	**Unaffected**	**HR**	**95% CI**	***p*-value**	**HR**	**95% CI**	***p*-value**
Q1 ref <9.44	11(1.9%)	550(98.1%)	—	—	—	—	—	—
Q2 9.44-12.58	21(3.8%)	522(96.2%)	1.64	0.79-3.42	0.18	1.63	0.77-3.42	0.19
Q3 12.59-17.16	25(4.7%)	509(95.3%)	1.88	0.92-3.83	0.08	1.85	0.88-3.89	0.10
Q4 >17.16	31(5.8%)	501(94.2%)	2.20	1.10-4.39	0.025	2.09	1.001-4.37	0.049

***Adjusted to (age, smoking, 1st degree relatives, adnexectomy, oral contraception, hormone replacement therapy)**

Table S.39 Hazard ratios for breast cancer by blood Pb level irrespective on age genotype MKI AA and APOB nonGG (quartiles).

			Univariate COX Regression			Multivariate COX Regression *		
**Blood Pb level** μg/L	**Cases**	**Unaffected**	**HR**	**95% CI**	***p*-value**	**HR**	**95% CI**	***p*-value**
Q1 <9.44	5(2.1%)	230(97.9%)	1.44	0.38-5.40	0.58	1.446	0.38-5.47	0.58
Q2 ref 9.44-12.58	4(1.7%)	221(98.3%)	—	—	—	—	—	—
Q3 12.59-17.16	15(7%)	199(93%)	3.69	1.22-11.12	0.02	3.98	1.29-12.22	0.015
Q4 >17.16	13(6%)	204(94%)	2.97	0.96-9.13	0.056	3.34	1.069-10.47	0.038

***Adjusted to (age, smoking, 1st degree relatives, adnexectomy, oral contraception, hormone replacement therapy)**

Table S.40 Hazard ratios for breast cancer by blood Pb level irrespective on age genotype MKI nonAA or APOB GG (quartiles).

			Univariate COX Regression			Multivariate COX Regression *		
**Blood Pb level** µg/L	**Cases**	**Unaffected**	**HR**	**95% CI**	***p*-value**	**HR**	**95% CI**	***p*-value**
Q1 <9.44	10(2.2%)	435(97.8%)	1.001	0.43-2.29	0.99	1.029	0.43-2.41	0.94
Q2 9.44-12.58	24(5.4%)	421(94.6%)	2.08	1.06-4.09	0.033	2.035	1.026-4.036	0.04
Q3 ref 12.59-17.16	13(2.9%)	432(97.1%)	—	—	—	—	—	—
Q4 >17.16	22(4.8%)	430(95.2%)	1.65	0.83-3.28	0.15	1.48	0.74-2.98	0.26

***Adjusted to (age, smoking, 1st degree relatives, adnexectomy, oral contraception, hormone replacement therapy)**

Table S.41 Hazard ratios for breast cancer by blood Pb level irrespective on age genotype MKI nonAA and APOB GG (quartiles).

			Univariate COX Regression			Multivariate COX Regression *		
**Blood Pb level** µg/L	**Cases**	**Unaffected**	**HR**	**95% CI**	***p*-value**	**HR**	**95% CI**	***p*-value**
Q1 <9.44	4(3.3%)	115(96.7%)	1.86	0.41-8.42	0.42	1.91	0.40-9.15	0.41
Q2 9.44-12.58	7(5.5%)	120(94.5%)	2.87	0.73-11.18	0.12	2.76	0.69-11.08	0.149
Q3 ref 12.59-17.16	3(2.4%)	122(97.6%)	—	—	—	—	—	—
Q4 >17.16	4(2.9%)	133(97.1%)	1.32	0.29-5.93	0.71	1.25	0.27-5.68	0.77

***Adjusted to (age, smoking, 1st degree relatives, adnexectomy, oral contraception, hormone replacement therapy)**

Table S.42 Hazard ratios for any cancer by blood Pb level for women above 50 years of age group genotype APOB nonGG or MKI nonAA (quartiles).

			Univariate COX Regression			Multivariate COX Regression *		
**Blood Pb level** µg/L	**Cases**	**Unaffected**	**HR**	**95% CI**	***p*-value**	**HR**	**95% CI**	***p*-value**
Q1 <9.44	17(11.5%)	130(88.5%)	1.72	0.95-3.10	0.07	1.72	0.94-3.13	0.07
Q2 9.44-12.58	21(8.4%)	229(91.6%)	1.20	0.69-2.08	0.51	1.19	0.68-2.07	0.53
Q3 12.59-17.16	36(10.4%)	308(89.6%)	1.33	0.83-2.15	0.23	1.34	0.83-2.18	0.22
Q4 ref >17.16	32(8%)	366(92%)	—	—	—	—	—	—

***Adjusted to (smoking, 1st degree relatives, adnexectomy, oral contraception, hormone replacement therapy)**

Table S.43 Hazard ratios for any cancer by blood Pb level for women above 50 years of age group genotype APOB nonGG and MKI nonAA (quartiles).

			Univariate COX Regression			Multivariate COX Regression *		
**Blood Pb level** µg/L	**Cases**	**Unaffected**	**HR**	**95% CI**	***p*-value**	**HR**	**95% CI**	***p*-value**
Q1 <9.44	4(7.5%)	49(92.5%)	1.79	0.48-6.71	0.38	1.93	0.50-7.37	0.33
Q2 9.44-12.58	8(11.7%)	60(88.3%)	2.73	0.89-8.37	0.07	2.89	0.93-8.91	0.06
Q3 12.59-17.16	9(9.8%)	83(90.2%)	2.13	0.71-6.37	0.17	2.45	0.80-7.43	0.11
Q4 ref >17.16	5(4.9%)	96(95.1%)	—	—	—	—	—	—

***Adjusted to (smoking, 1st degree relatives, adnexectomy, oral contraception, hormone replacement therapy)**

Table S.44 Hazard ratios for any cancer by blood Pb level for women above 50 years of age group genotype APOB GG or MKI AA (quartiles).

			Univariate COX Regression			Multivariate COX Regression *		
**Blood Pb level** µg/L	**Cases**	**Unaffected**	**HR**	**95% CI**	***p*-value**	**HR**	**95% CI**	***p*-value**
Q1 <9.44	15(10.4%)	129(89.6%)	1.35	0.71-2.54	0.35	1.34	0.70-2.53	0.36
Q2 ref 9.44-12.58	26(8.8%)	268(91.2%)	—	—	—	—	—	—
Q3 12.59-17.16	39(9.9%)	352(90.1%)	1.04	0.63-1.71	0.86	1.03	0.62-1.71	0.88
Q4 >17.16	43(9.6%)	404(90.4%)	1.12	0.69-1.80	0.63	1.11	0.68-1.79	0.66

***Adjusted to (smoking, 1st degree relatives, adnexectomy, oral contraception, hormone replacement therapy)**

Table S.45 Hazard ratios for any cancer by blood Pb level for women above 50 years of age group genotype APOB GG and MKI AA (quartiles).

			Univariate COX Regression			Multivariate COX Regression *		
**Blood Pb level** µg/L	**Cases**	**Unaffected**	**HR**	**95% CI**	***p*-value**	**HR**	**95% CI**	***p*-value**
Q1 ref <9.44	2(4%)	48(96%)	—	—	—	—	—	—
Q2 9.44-12.58	13(11.6%)	99(88.4%)	2.15	0.48-9.58	0.31	2.19	0.49-9.83	0.30
Q3 12.59-17.16	12(8.6%)	127(91.4%)	1.64	0.36-7.38	0.51	1.71	0.37-7.80	0.48
Q4 >17.16	22(14%)	134(86%)	2.71	0.63-11.56	0.17	2.70	0.62-11.62	0.18

***Adjusted to (smoking, 1st degree relatives, adnexectomy, oral contraception, hormone replacement therapy)**

Table S.46 Hazard ratios for any cancer by blood Pb level for women above 50 years of age group genotype MKI AA or APOB nonGG (quartiles).

			Univariate COX Regression			Multivariate COX Regression *		
**Blood Pb level** μg/L	**Cases**	**Unaffected**	**HR**	**95% CI**	***p*-value**	**HR**	**95% CI**	***p*-value**
Q1 <9.44	14(8.5%)	149(91.5%)	1.025	0.56-1.86	0.93	1.04	0.56-1.91	0.89
Q2 9.44-12.58	29(9.6%)	273(90.4%)	1.017	0.63-1.62	0.94	1.013	0.63-1.62	0.95
Q3 12.59-17.16	42(10.6%)	352(89.4%)	1.095	0.72-1.66	0.67	1.10	0.72-1.69	0.64
Q4 ref >17.16	45(10%)	396(90%)	—	—	—	—	—	—

***Adjusted to (smoking, 1st degree relatives, adnexectomy, oral contraception, hormone replacement therapy)**

Table S.47 Hazard ratios for any cancer by blood Pb level for women above 50 years of age group genotype MKI AA and APOB nonGG (quartiles).

			Univariate COX Regression			Multivariate COX Regression *		
**Blood Pb level** μg/L	**Cases**	**Unaffected**	**HR**	**95% CI**	***p*-value**	**HR**	**95% CI**	***p*-value**
Q1 <9.44	8(13.3%)	52(86.7%)	2.21	0.83-5.90	0.11	2.18	0.81-5.86	0.12
Q2 ref 9.44-12.58	8(6.5%)	114(93.5%)	—	—	—	—	—	—
Q3 12.59-17.16	21(12.8%)	142(87.2%)	1.86	0.82-4.21	0.13	1.90	0.84-4.33	0.12
Q4 >17.16	18(9.7%)	166(90.3%)	1.33	0.57-3.06	0.50	1.39	0.60-3.24	0.43

***Adjusted to (smoking, 1st degree relatives, adnexectomy, oral contraception, hormone replacement therapy)**

Table S.48 Hazard ratios for any cancer by blood Pb level for women above 50 years of age group genotype MKI nonAA or APOB GG (quartiles).

			Univariate COX Regression			Multivariate COX Regression *		
**Blood Pb level** µg/L	**Cases**	**Unaffected**	**HR**	**95% CI**	***p*-value**	**HR**	**95% CI**	***p*-value**
Q1 <9.44	11(8%)	126(92%)	1.17	0.58-2.37	0.65	1.17	0.57-2.39	0.65
Q2 9.44-12.58	26(10.8%)	214(89.2%)	1.39	0.81-2.38	0.23	1.37	0.79-2.37	0.25
Q3 ref 12.59-17.16	27(8.4%)	293(91.6%)	—	—	—	—	—	—
Q4 >17.16	36(9.7%)	334(90.3%)	1.15	0.70-1.90	0.57	1.12	0.67-1.86	0.65

***Adjusted to (smoking, 1st degree relatives, adnexectomy, oral contraception, hormone replacement therapy)**

Table S.49 Hazard ratios for any cancer by blood Pb level for women above 50 years of age group genotype MKI nonAA and APOB GG (quartiles).

			Univariate COX Regression			Multivariate COX Regression *		
**Blood Pb level** µg/L	**Cases**	**Unaffected**	**HR**	**95% CI**	***p*-value**	**HR**	**95% CI**	***p*-value**
Q1 <9.44	5(14.7%)	29(85.3%)	2.86	0.86-9.44	0.08	2.85	0.85-9.55	0.09
Q2 9.44-12.58	5(8.3%)	55(91.7%)	1.58	0.48-5.25	0.45	1.45	0.43-4.90	0.54
Q3 ref 12.59-17.16	6(6.7%)	83(93.3%)	—	—	—	—	—	—
Q4 >17.16	9(7.9%)	104(92.1%)	1.25	0.44-3.52	0.66	1.26	0.44-3.59	0.65

***Adjusted to (smoking, 1st degree relatives, adnexectomy, oral contraception, hormone replacement therapy)**

Table S.50 Hazard ratios for breast cancer by blood Pb level on women above 50 years of age genotype APOB nonGG or MKI nonAA (quartiles).

			Univariate COX Regression			Multivariate COX Regression *		
**Blood Pb level** μg/L	**Cases**	**Unaffected**	**HR**	**95% CI**	***p*-value**	**HR**	**95% CI**	***p*-value**
Q1 <9.44	5(0.9%)	513(99.1%)	1.40	0.54-3.66	0.48	1.56	0.58-4.12	0.37
Q2 9.44-12.58	6(1.1%)	517(98.9%)	1.18	0.51-2.74	0.69	1.22	0.52-2.84	0.64
Q3 12.59-17.16	16(3.1%)	498(96.9%)	1.53	0.76-3.078	0.23	1.64	0.81-3.33	0.16
Q4 ref >17.16	29(5.3%)	513(94.7%)	—	—	—	—	—	—

***Adjusted to (smoking, 1st degree relatives, adnexectomy, oral contraception, hormone replacement therapy)**

Table S.51 Hazard ratios for breast cancer by blood Pb level on women above 50 years of age genotype APOB nonGG and MKI nonAA (quartiles).

			Univariate COX Regression			Multivariate COX Regression *		
**Blood Pb level** μg/L	**Cases**	**Unaffected**	**HR**	**95% CI**	***p*-value**	**HR**	**95% CI**	***p*-value**
Q1 <9.44	2(3.9%)	49(96.1%)	—	—	—	—	—	—
Q2 9.44-12.58	6(9%)	60(91%)	—	—	—	—	—	—
Q3 12.59-17.16	4(4.6%)	83(95.4%)	—	—	—	—	—	—
Q4 >17.16	0	96	—	—	—	—	—	—

*Adjusted to (smoking, 1st degree relatives, adnexectomy, oral contraception, hormone replacement therapy)

Table S.52 Hazard ratios for breast cancer by blood Pb level on women above 50 years of age genotype APOB GG or MKI AA (quartiles).

			Univariate COX Regression			Multivariate COX Regression *		
**Blood Pb level** µg/L	**Cases**	**Unaffected**	**HR**	**95% CI**	***p*-value**	**HR**	**95% CI**	***p*-value**
Q1 <9.44	6(4.4%)	130(95.6%)	1.96	0.59-6.42	0.26	2.04	0.62-6.71	0.24
Q2 ref 9.44-12.58	9(3.8%)	229(96.2%)	—	—	—	—	—	—
Q3 12.59-17.16	18(5.5%)	308(94.5%)	1.85	0.72-4.74	0.19	1.92	0.74-4.94	0.17
Q4 >17.16	14(3.6%)	366(96.4%)	2.86	1.18-6.89	0.019	2.82	1.16-6.83	0.021

*Adjusted to (smoking, 1st degree relatives, adnexectomy, oral contraception, hormone replacement therapy)

Table S.53 Hazard ratios for breast cancer by blood Pb level on women above 50 years of age genotype MKI AA or APOB nonGG (quartiles).

			Univariate COX Regression			Multivariate COX Regression *		
**Blood Pb level** µg/L	**Cases**	**Unaffected**	**HR**	**95% CI**	***p*-value**	**HR**	**95% CI**	***p*-value**
Q1 <9.44	5(3.2%)	149(96.8%)	1.03	0.35-3.027	0.95	1.06	0.36-3.13	0.90
Q2 ref 9.44-12.58	10(3.5%)	273(96.5%)	—	—	—	—	—	—
Q3 12.59-17.16	17(4.6%)	352(95.4%)	1.25	0.57-2.73	0.57	1.29	0.58-2.84	0.52

Q4 >17.16	26(6.1%)	396(93.9%)	1.62	0.78-3.36	0.19	1.59	0.76-3.32	0.21

***Adjusted to (smoking, 1st degree relatives, adnexectomy, oral contraception, hormone replacement therapy)**

Table S.54 Hazard ratios for breast cancer by blood Pb level on women above 50 years of age genotype MKI AA and APOB nonGG (quartiles).

			Univariate COX Regression			Multivariate COX Regression *		
**Blood Pb level** µg/L	**Cases**	**Unaffected**	**HR**	**95% CI**	***p*-value**	**HR**	**95% CI**	***p*-value**
Q1 <9.44	2(3.7%)	52(96.3%)	4.57	0.41-50.48	0.21	4.46	0.40-49.74	0.22
Q2 ref 9.44-12.58	1(0.8%)	114(99.2%)	—	—	—	—	—	—
Q3 12.59-17.16	11(7.2%)	142(92.8%)	7.93	1.02-61.48	0.047	8.79	1.12-68.62	0.038
Q4 >17.16	11(6.2%)	166(93.8%)	6.57	0.84-50.94	0.07	7.78	0.99-60.87	0.05

***Adjusted to (smoking, 1st degree relatives, adnexectomy, oral contraception, hormone replacement therapy)**

Table S.55 Hazard ratios for breast cancer by blood Pb level on women above 50 years of age genotype MKI nonAA or APOB GG (quartiles).

			Univariate COX Regression			Multivariate COX Regression *		
**Blood Pb level** µg/L	**Cases**	**Unaffected**	**HR**	**95% CI**	***p*-value**	**HR**	**95% CI**	***p*-value**
Q1 <9.44	5(3.8%)	126(96.2%)	1.59	0.53-4.78	0.40	1.78	0.59-5.41	0.30
Q2 9.44-12.58	11(4.8%)	214(95.2%)	1.80	0.74-4.35	0.18	1.68	0.68-4.098	0.25

Q3 ref 12.59-17.16	9(2.9%)	293(97.1%)	—	—	—	—	—	—
Q4 >17.16	18(5.1%)	334(94.9%)	1.72	0.77-3.84	0.18	1.50	0.66-3.39	0.32

***Adjusted to (smoking, 1st degree relatives, adnexectomy, oral contraception, hormone replacement therapy)**

Table S.56 Hazard ratios for breast cancer by blood Pb level on women above 50 years of age genotype MKI nonAA and APOB GG (quartiles).

			Univariate COX Regression			Multivariate COX Regression *		
**Blood Pb level** µg/L	**Cases**	**Unaffected**	**HR**	**95% CI**	***p*-value**	**HR**	**95% CI**	***p*-value**
Q1 <9.44	2(6.4%)	29(93.6%)	2.78	0.46-16.80	0.26	4.42	0.65-29.69	0.12
Q2 9.44-12.58	2(3.5%)	55(96.5%)	1.49	0.24-9.01	0.66	1.37	0.22-8.44	0.73
Q3 12.59-17.16	3(3.6%)	83(96.4%)	1.16	0.23-5.78	0.85	1.46	0.28-7.42	0.64
Q4 ref >17.16	3(2.8%)	104(97.2%)	—	—	—	—	—	—

***Adjusted to (smoking, 1st degree relatives, adnexectomy, oral contraception, hormone replacement therapy)**

Table S.57 Hazard ratios for any cancer by blood Pb level for women below 50 years of age group genotype APOB nonGG or MKI nonAA (quartiles).

			Univariate COX Regression			Multivariate COX Regression *		
**Blood Pb level** µg/L	**Cases**	**Unaffected**	**HR**	**95% CI**	***p*-value**	**HR**	**95% CI**	***p*-value**
Q1 ref <9.44	9(2.4%)	367(97.6%)	—	—	—	—	—	—

Q2 9.44-12.58	17(6.7%)	237(93.3%)	2.52	1.12-5.68	0.025	2.51	1.12-5.66	0.025
Q3 12.59-17.16	12(7.6%)	146(92.4%)	2.53	1.062-6.021	0.036	2.57	1.07-6.14	0.033
Q4 >17.16	6(6.1%)	92(93.9%)	2.11	0.75-5.96	0.16	2.10	0.74-5.95	0.16

***Adjusted to (smoking, 1st degree relatives, adnexectomy, oral contraception, hormone replacement therapy)**

Table S.58 Hazard ratios for any cancer by blood Pb level for women below 50 years of age group genotype APOB nonGG and MKI nonAA (quartiles).

			Univariate COX Regression			Multivariate COX Regression *		
**Blood Pb level** µg/L	**Cases**	**Unaffected**	**HR**	**95% CI**	***p*-value**	**HR**	**95% CI**	***p*-value**
Q1 <9.44	2(1.9%)	103(98.1%)	—	—	—	—	—	—
Q2 9.44-12.58	7(9.7%)	65(90.3%)	5.28	1.09-25.46	0.037	5.22	1.07-25.32	0.04
Q3 12.59-17.16	5(9%)	50(91%)	4.34	0.84-22.38	0.07	3.52	0.65-18.95	0.14
Q4 >17.16	1(3.8%)	25(96.2%)	1.64	0.15-18.21	0.68	1.17	0.10-13.79	0.89

***Adjusted to (smoking, 1st degree relatives, adnexectomy, oral contraception, hormone replacement therapy)**

Table S.59 Hazard ratios for any cancer by blood Pb level for women below 50 years of age group genotype APOB GG or MKI AA (quartiles).

			Univariate COX Regression			Multivariate COX Regression *		
**Blood Pb level** µg/L	**Cases**	**Unaffected**	**HR**	**95% CI**	***p*-value**	**HR**	**95% CI**	***p*-value**

Q1 ref <9.44	10(2.5%)	384(97.5%)	—	—	—	—	—	—
Q2 9.44-12.58	12(4.6%)	249(95.4%)	1.52	0.65-3.53	0.32	1.59	0.68-3.72	0.27
Q3 12.59-17.16	11(7%)	146(93%)	2.14	0.90-5.06	0.08	2.20	0.92-5.23	0.07
Q4 >17.16	7(6%)	109(94%)	1.90	0.72-5.023	0.19	2.058	0.77-5.47	0.14

***Adjusted to (smoking, 1st degree relatives, adnexectomy, oral contraception, hormone replacement therapy)**

Table S.60 Hazard ratios for any cancer by blood Pb level for women below 50 years of age group genotype APOB GG and MKI AA (quartiles).

			Univariate COX Regression			Multivariate COX Regression *		
**Blood Pb level** µg/L	**Cases**	**Unaffected**	**HR**	**95% CI**	***p*-value**	**HR**	**95% CI**	***p*-value**
Q1 <9.44	3(2.4%)	120(97.6%)	1.25	0.20-7.52	0.80	1.036	0.16-6.56	0.96
Q2 ref 9.44-12.58	2(2.5%)	77(97.5%)	—	—	—	—	—	—
Q3 12.59-17.16	4(7.4%)	50(92.6%)	3.27	0.59-17.91	0.17	2.95	0.52-16.53	0.22
Q4 >17.16	2(4.5%)	42(95.5%)	1.66	0.23-11.93	0.61	2.36	0.28-19.65	0.42

***Adjusted to (smoking, 1st degree relatives, adnexectomy, oral contraception, hormone replacement therapy)**

Table S.61 Hazard ratios for any cancer by blood Pb level for women below 50 years of age group genotype MKI AA or APOB nonGG (quartiles).

			Univariate COX Regression			Multivariate COX Regression *		
**Blood Pb level** µg/L	**Cases**	**Unaffected**	**HR**	**95% CI**	***p*-value**	**HR**	**95% CI**	***p*-value**
Q1 ref <9.44	8(1.9%)	401(98.1%)	—	—	—	—	—	—
Q2 9.44-12.58	14(5.3%)	249(94.7%)	2.33	0.98-5.58	0.05	2.37	0.99-5.69	0.05
Q3 12.59-17.16	15(8.7%)	157(91.3%)	3.81	1.61-9.01	0.002	3.83	1.61-9.10	0.002
Q4 >17.16	7(6.2%)	105(93.8%)	2.47	0.89-6.84	0.08	2.55	0.90-7.17	0.076

***Adjusted to (smoking, 1st degree relatives, adnexectomy, oral contraception, hormone replacement therapy)**

Table S.62 Hazard ratios for any cancer by blood Pb level for women below 50 years of age group genotype MKI AA and APOB nonGG (quartiles).

			Univariate COX Regression			Multivariate COX Regression *		
**Blood Pb level** µg/L	**Cases**	**Unaffected**	**HR**	**95% CI**	***p*-value**	**HR**	**95% CI**	***p*-value**
Q1 ref <9.44	3(1.6%)	178(98.4%)	—	—	—	—	—	—
Q2 9.44-12.58	5(4.4%)	107(95.6%)	2.21	0.52-9.31	0.27	2.19	0.52-9.24	0.28
Q3 12.59-17.16	6(9.5%)	57(90.5%)	4.59	1.14-18.47	0.03	4.75	1.17-19.32	0.029
Q4 >17.16	4(9.5%)	38(90.5%)	4.39	0.97-19.80	0.05	4.18	0.91-19.13	0.06

***Adjusted to (smoking, 1st degree relatives, adnexectomy, oral contraception, hormone replacement therapy)**

Table S.63 Hazard ratios for any cancer by blood Pb level for women below 50 years of age group genotype MKI nonAA or APOB GG (quartiles).

			Univariate COX Regression			Multivariate COX Regression *		
**Blood Pb level** µg/L	**Cases**	**Unaffected**	**HR**	**95% CI**	***p*-value**	**HR**	**95% CI**	***p*-value**
Q1 ref <9.44	9(2.8%)	309(97.2%)	—	—	—	—	—	—
Q2 9.44-12.58	14(6.3%)	207(94.7%)	1.99	0.86-4.63	0.10	2.01	0.86-4.69	0.10
Q3 12.59-17.16	10(6.7%)	139(93.3%)	1.93	0.78-4.78	0.15	1.94	0.78-4.84	0.15
Q4 >17.16	4(4%)	96(96%)	1.17	0.35-3.81	0.79	1.14	0.34-3.82	0.82

***Adjusted to (smoking, 1st degree relatives, adnexectomy, oral contraception, hormone replacement therapy)**

Table S.64 Hazard ratios for any cancer by blood Pb level for women below 50 years of age group genotype MKI nonAA and APOB GG (quartiles).

			Univariate COX Regression			Multivariate COX Regression *		
**Blood Pb level** µg/L	**Cases**	**Unaffected**	**HR**	**95% CI**	***p*-value**	**HR**	**95% CI**	***p*-value**
Q1 <9.44	4(4.4%)	86(95.6%)	2.69	0.29-24.59	0.38	2.49	0.27-22.86	0.41
Q2 9.44-12.58	5(7.1%)	65(92.9%)	3.98	0.46-34.35	0.20	3.80	0.44-32.88	0.22
Q3 ref 12.59-17.16	1(2.5%)	39(97.5%)	—	—	—	—	—	—
Q4 >17.16	1(3.3%)	29(96.7%)	1.87	0.11-30.23	0.65	1.51	0.09-25.32	0.77

***Adjusted to (smoking, 1st degree relatives, adnexectomy, oral contraception, hormone replacement therapy)**

Table S.65 Hazard ratios for breast cancer by blood Pb level for genotype APOB nonGG or MKI nonAA below 50 years of age (quartiles).

			Univariate COX Regression			Multivariate COX Regression *		
**Blood Pb level** μg/L	**Cases**	**Unaffected**	**HR**	**95% CI**	***p*-value**	**HR**	**95% CI**	***p*-value**
Q1 ref <9.44	5(1.3%)	367 (98.7%)	—	—	—	—	—	—
Q2 9.44-12.58	14(5.5%)	237(94.5%)	3.75	1.35-10.45	0.011	3.68	1.32-10.27	0.012
Q3 12.59-17.16	7(4.5%)	146(95.5%)	2.76	0.87-8.72	0.08	2.61	0.82-8.32	0.10

Q4 >17.16	4(4.2%)	92(95.8%)	2.57	0.68-9.60	0.16	2.39	0.63-8.99	0.19
Q1 vs Q2-4								
<9.44	6(1.6%)	366(98.4%)						
>9.44	25(5%)	475(95%)	2.62	1.07-6.40	0.035	2.53	1.032-6.23	0.041

***Adjusted to (smoking, 1st degree relatives, adnexectomy, oral contraception, , hormone replacement therapy)**

Table S.66 Hazard ratios for breast cancer by blood Pb level on women below 50 years of age genotype APOB nonGG and MKI nonAA (quartiles).

			Univariate COX Regression			Multivariate COX Regression *		
**Blood Pb level** µg/L	**Cases**	**Unaffected**	**HR**	**95% CI**	***p*-value**	**HR**	**95% CI**	***p*-value**
Q1 ref <9.44	1(0.9%)	102(99.1%)	—	—	—	—	—	—
Q2 9.44-12.58	6(8.4%)	65(91.2%)	8.51	1.02-70.82	0.047	7.35	0.86-62.29	0.067
Q3 12.59-17.16	3(5.6%)	50(94.4%)	5.39	0.56-51.90	0.14	3.54	0.35-35.87	0.28

Q4 >17.16	1(3.8%)	25(96.2%)	3.46	0.21-56.07	0.38	1.89	0.11-32.63	0.66

***Adjusted to (smoking, 1stdegree relatives, adnexectomy, oral contraception, hormone replacement therapy)**

Table S.67 Hazard ratios for breast cancer by blood Pb level on women below 50 years of age genotype APOB GG or MKI AA (quartiles).

			Univariate COX Regression			Multivariate COX Regression *		
**Blood Pb level** μg/L	**Cases**	**Unaffected**	**HR**	**95% CI**	***p*-value**	**HR**	**95% CI**	***p*-value**
Q1 ref <9.44	8(2%)	384(98%)	—	—	—	—	—	—
Q2 9.44-12.58	10(3.8%)	249(96.2%)	1.57	0.61-4.00	0.34	1.73	0.25	0.67-4.45
Q3 12.59-17.16	5(3.3%)	146(96.7%)	1.23	0.40-3.78	0.71	1.27	0.67	0.41-3.95
Q4 >17.16	5(4.4%)	109(95.6%)	1.66	0.54-5.13	0.37	1.76	0.32	0.56-5.47

***Adjusted to (smoking, 1stdegree relatives, adnexectomy, oral contraception, hormone replacement therapy)**

Table S.68 Hazard ratios for breast cancer by blood Pb level on women below 50 years of age genotype APOB GG and MKI AA (quartiles).

			Univariate COX Regression			Multivariate COX Regression *		
**Blood Pb level** µg/L	**Cases**	**Unaffected**	**HR**	**95% CI**	***p*-value**	**HR**	**95% CI**	***p*-value**
Q1 <9.44	3(2.4%)	120(97.6%)	1.52	0.15-14.71	0.71	1.28	0.13-12.45	0.83
Q2 9.44-12.58	2(2.5%)	77(97.5%)	1.15	0.61-4.00	0.90	1.25	0.10-14.44	0.85
Q3 ref 12.59-17.16	1(1.9%)	50(98.1%)	—	—	—	—	—	—
Q4 >17.16	2(4.5%)	42(95.5%)	1.88	0.16-21.13	0.60	3.32	0.22-48.25	0.38

***Adjusted to (smoking, 1st degree relatives, adnexectomy, oral contraception, hormone replacement therapy)**

Table S.69 Hazard ratios for breast cancer by blood Pb level on women below 50 years of age genotype MKI AA or APOB nonGG (quartiles).

			Univariate COX Regression			Multivariate COX Regression *		
**Blood Pb level** µg/L	**Cases**	**Unaffected**	**HR**	**95% CI**	***p*-value**	**HR**	**95% CI**	***p*-value**
Q1 ref <9.44	6(14.7%)	401(85.3%)	—	—	—	—	—	—
Q2 9.44-12.58	11(4.2%)	249(95.8%)	2.60	1.11-6.11	0.027	2.65	1.13-6.24	0.025
Q3 12.59-17.16	8(4.8%)	157(95.2%)	1.96	0.73-5.24	0.18	1.93	0.72-5.18	0.19
Q4 >17.16	5(4.5%)	105(95.5%)	2.11	0.73-6.12	0.16	2.02	0.69-5.90	0.19

***Adjusted to (smoking, 1st degree relatives, adnexectomy, oral contraception, hormone replacement therapy)**

Table S.70 Hazard ratios for breast cancer by blood Pb level on women below 50 years of age genotype MKI AA and APOB nonGG (quartiles).

			Univariate COX Regression			Multivariate COX Regression *		
**Blood Pb level** μg/L	**Cases**	**Unaffected**	**HR**	**95% CI**	***p*-value**	**HR**	**95% CI**	***p*-value**
Q1 ref <9.44	3(1.6%)	178(98.4%)	—	—	—	—	—	—
Q2 9.44-12.58	3(2.7%)	107(97.3%)	1.24	0.24-6.20	0.79	1.26	0.25-6.35	0.77
Q3 12.59-17.16	4(6.5%)	57(93.5%)	2.93	0.65-13.19	0.16	2.71	0.58-12.51	0.20
Q4 >17.16	2(5%)	38(95%)	2.025	0.33-12.22	0.44	1.78	0.28-11.03	0.53

***Adjusted to (smoking, 1st degree relatives, adnexectomy, oral contraception, hormone replacement therapy)**

Table S.71 Hazard ratios for breast cancer by blood Pb level on women below 50 years of age genotype MKI nonAA or APOB GG (quartiles).

			Univariate COX Regression			Multivariate COX Regression *		
**Blood Pb level** μg/L	**Cases**	**Unaffected**	**HR**	**95% CI**	***p*-value**	**HR**	**95% CI**	***p*-value**
Q1 ref <9.44	5(1.6%)	309(98.4%)	—	—	—	—	—	—
Q2 9.44-12.58	13(5.9%)	207(94.1%)	3.35	1.19-9.43	0.02	3.27	1.15-9.28	0.025
Q3 12.59-17.16	4(2.8%)	139(97.2%)	1.47	0.39-5.51	0.56	1.35	0.35-5.12	0.65
Q4 >17.16	4(4%)	96(96%)	2.11	0.56-7.91	0.26	1.84	0.47-7.13	0.37

***Adjusted to (smoking, 1st degree relatives, adnexectomy, oral contraception, hormone replacement therapy)**

Table S.72 Hazard ratios for breast cancer by blood Pb level on women below 50 years of age genotype MKI nonAA and APOB GG (quartiles).

			Univariate COX Regression			Multivariate COX Regression *		
**Blood Pb level** μg/L	**Cases**	**Unaffected**	**HR**	**95% CI**	***p*-value**	**HR**	**95% CI**	***p*-value**
Q1 ref <9.44	2(2.2%)	86(98.4%)	—	—	—	—	—	—
Q2 9.44-12.58	5(7.1%)	65(94.1%)	3.22	0.62-16.64	0.16	3.37	0.65-17.48	0.14
Q3 12.59-17.16	0	39	—	—	—	—	—	—
Q4 >17.16	1(3.33%)	29(96.66%)	1.43	0.13-15.83	0.76	1.17	0.10-13.51	0.89

***Adjusted to (smoking, 1st degree relatives, adnexectomy, oral contraception, hormone replacement therapy)**
